# The Cognitive Profile of Math Difficulties: A Meta-Analysis Based on Clinical Criteria

**DOI:** 10.3389/fpsyg.2022.842391

**Published:** 2022-03-11

**Authors:** Stefan Haberstroh, Gerd Schulte-Körne

**Affiliations:** Department of Child and Adolescent Psychiatry, Psychosomatics and Psychotherapy, Ludwig-Maximilian-University of Munich, Munich, Germany

**Keywords:** math difficulties, dyscalculia, math disabilities, domain-general abilities, domain-specific abilities, meta-analysis, systematic review

## Abstract

Math difficulties (MD) manifest across various domain-specific and domain-general abilities. However, the existing cognitive profile of MD is incomplete and thus not applicable in typical settings such as schools or clinics. So far, no review has applied inclusion criteria according to DSM or ICD, summarized domain-specific abilities or examined the validity of response time scores for MD identification. Based upon stringent clinical criteria, the current meta-analysis included 34 studies which compared cognitive performances of a group with MD (*n* = 680) and a group without MD (*n* = 1565). Criteria according to DSM and ICD were applied to identify MD (percentile rank ≤ 16, age range 8–12 years, no comorbidities/low IQ). Effect sizes for 22 abilities were estimated and separated by their level and type of scoring (AC = accuracy, RT = response time). A cognitive profile of MD was identified, characterized by distinct weaknesses in: (a) computation (calculation [AC], fact retrieval [AC]), (b) number sense (quantity processing [AC], quantity-number linking [RT], numerical relations [AC]), and (c) visual-spatial short-term storage [AC]. No particular strength was found. Severity of MD, group differences in reading performance and IQ did not significantly moderate the results. Further analyses revealed that (a) effects are larger when dealing with numbers or number words than with quantities, (b) MD is not accompanied by any weakness in abilities typically assigned to reading, and (c) weaknesses in visual-spatial short-term storage emphasize the notion that number and space are interlinked. The need for high-quality studies investigating domain-general abilities is discussed.

## Introduction

About 3–6% of all children experience severe difficulties in mathematics despite having normal intelligence and access to adequate education (Shalev, 2007; Moll et al., 2014; Fortes et al., 2016). If not identified and treated at an early stage, math difficulties (MD) can persistently affect academic functioning (Shalev et al., 2005; Morgan et al., 2009) and increase the risk of mental health problems (Willcutt et al., 2013; Endlich et al., 2014; Devine et al., 2018).

Given these possible consequences, it is essential to identify MD correctly (Ritchie and Bates, 2013; Lewis and Fisher, 2016). Although different diagnostic approaches exist (e.g., response to intervention), in most cases MD is diagnosed when performance in cognitive abilities related to MD is below average. Usually DSM-5 or ICD-10 (soon ICD-11) is used for this kind of MD identification. However, both offer only a short description of cognitive abilities which are affected by MD. In fact, a valid cognitive profile of MD based on clinical criteria in accordance with DSM or ICD is still missing (Geary, 2010; Pham and Riviere, 2015; Träff et al., 2017).

What is known is that MD manifest across several mathematical (e.g., fact retrieval) and also non-mathematical (e.g., working memory) abilities, which are also referred to as domain-specific abilities and domain-general abilities, respectively (Henik et al., 2011). While prior reviews have reported a huge variety of strengths and weaknesses in those abilities (e.g., Cowan and Powell, 2014; Karagiannakis et al., 2014; Shin and Bryant, 2015; Peng et al., 2018), they have not applied inclusion criteria to identify MD which are in accordance with diagnostic procedures given by DSM and ICD. In most cases, inclusion criteria were too liberal (e.g., high cut-off value, age range too broad) or group differences between people with and without MD were not controlled for (e.g., comorbid reading difficulties). In addition, important parts to complete the overall cognitive profile of MD have never been systematically reviewed yet. Most importantly, no meta-analysis has summarized domain-specific abilities or has systematically considered different levels of abilities. Especially number sense, which refers to the basic processing of magnitudes and numbers, consists of multiple sub-abilities (Butterworth et al., 2011; Kaufmann and von Aster, 2012; Hirsch et al., 2018). Although DSM and ICD suggest to measure number sense for MD identification, it is unclear how each sub-ability (e.g., quantity processing) differs between people with and without MD. Also, most studies have focused only on abilities which were measured by using accuracy scores (e.g., number of solved items) while response time scores are clearly under researched and not discussed in terms of their validity to differentiate between people with and without MD (e.g., Hanich et al., 2001; Mammarella et al., 2013).

Therefore, the aim of this review is to fill these research gaps and to define a most comprehensive cognitive profile of MD based on clinical criteria given by DSM and ICD. For this reason, we applied strict inclusion criteria, differentiated several ability levels and compared various scoring types to make the most precise statements about strengths and weaknesses in MD.

The manuscript is structured as follows: In the first part of the introduction we summarize the results of prior reviews, while in the second part we relate these results to the aims of our analysis. After describing the methods and results of our study we discuss how and why our cognitive profile of MD differs from existing results and how it is related to existing cognitive theories and neurobiological studies about MD.

### Prior Reviews and Meta-Analysis

#### Domain-Specific Abilities

According to DSM and ICD MD is described by weaknesses in computation, math reasoning and number sense (American Psychiatric Association, 2013; World Health Organization, 2018). These weaknesses are also referred to as domain-specific weaknesses, since they are all directly related to mathematics (Henik et al., 2011).

##### Computation and Math Reasoning

Computation means to solve arithmetical problems. Depending on the type of arithmetical problem, computation can be divided into two sub-abilities: Fact retrieval and calculation. Fact retrieval is used when simple arithmetical problems (e.g., multiplication tables) are solved by retrieval of facts from long-term memory. Calculation is used when dealing with more complex and multi-step problems (e.g., 352 + 943), which require an overall understanding of basic arithmetic operations (Busch et al., 2015). Both calculation and fact retrieval are considered as the hallmark symptoms of MD, as deficits in these abilities are most prevalent (Jordan and Hanich, 2003). However, that is not the case with math reasoning and number sense which still lack a consistent definition. As a consequence, both abilities are measured in various ways using different tasks, which in turn, lead to different strengths and weaknesses in people with MD (Berch, 2005; Gersten et al., 2005).

Math reasoning is understood as the application of mathematical knowledge to solve unfamiliar problems (Lindquist et al., 2017). Although several tasks to measure math reasoning exist, no clear differentiation in terms of sub-abilities can be made. Based on the description of math reasoning above, two groups of tasks can be separated. The first group consists of tasks which require participants to solve rather complex problems by using their full mathematical knowledge (Kaufmann and von Aster, 2012; Casey et al., 2015). A typical task for this group is word problems, which is often used to measure math reasoning and also leads to large deficits in people with MD (Yip et al., 2020). In addition, tasks which involve the interpretation of data (e.g., tables and charts) or geometry have been applied too (Proctor, 2012; Tolar et al., 2016). The second group includes tasks which focus more on reasoning than on knowledge. For example, Zhang et al. (2017) used number series (i.e., find a pattern in a given sequence of numbers by applying basic arithmetic operations) in addition to word problems to measure math reasoning. However, number series tasks correlate strongly with fluid reasoning (i.e., non-verbal IQ) so that deficits in math reasoning could be moderated more by general IQ than by MD status (Floyd et al., 2003; Benson et al., 2016). But no studies exist which compare both type of tasks in people with MD.

##### Number Sense

Certainly, the most difficult domain-specific ability to pinpoint and to measure is number sense. Number sense in general refers to difficulties in processing magnitudes and numbers (Butterworth et al., 2011). However, several sub-abilities have been assigned to number sense in the past (for an overview: Kaufmann and von Aster, 2012; Hirsch et al., 2018). These sub-abilities can be differentiated in terms of what type of information is processed and what type of cognitive task is demanded as follows: (1) knowing numerals (e.g., counting aloud, transcoding), (2) processing quantities (e.g., non-symbolic comparison), (3) linking quantities to numerals/numbers (e.g., subitizing and dot enumeration), and (4) relating numbers (e.g., symbolic comparison, number line). This sequence (from numerals and quantities to numbers) is also in line with various numerical development models (Krajewski and Schneider, 2009; Michalczyk et al., 2013; Simanowski and Krajewski, 2019). Another approach would be to separate between processing non-symbolic numerosities (e.g., set of dots), symbolic numbers (e.g., digits) and the mapping between both (Kolkman et al., 2013; Huijsmans et al., 2020). Although longitudinal studies have shown that number sense in kindergarten is strongly predictive of math performance in school, people with MD show different strengths and weaknesses depending on which number sense sub-ability is measured (Jordan et al., 2009; Desoete et al., 2012; Geary et al., 2012). Currently only one meta-analysis examines differences between people with and without MD in number sense, which is also the only published meta-analysis for domain-specific abilities in general. Based on 19 studies, Schwenk et al. (2017) reported a more severe weakness in processing of numbers (symbolic comparison) than of quantities (non-symbolic comparison). Effect size for quantity processing was 0.24, while the effect size for number processing was 0.75. This result is in line with a meta-analysis by Schneider et al. (2017) who reported a higher correlation of math performance with number than quantity processing in people without MD. Regarding the simultaneously processing of quantities and numbers, results are still ambiguous. Overall, people with MD do not always show a consistent weakness in dot enumeration or subitizing tasks (Landerl, 2013; Szûcs et al., 2013; Skagerlund and Träff, 2014). Especially for subitizing there are still a lot of unsolved questions, for example, whether subitizing even belongs to mathematics or whether it is rather a general cognitive mechanism to process different kinds of magnitudes (e.g., space and time) (Anobile et al., 2019). However, a robust weakness seems to be prevalent in people with MD when dealing with numerals, especially in transcoding (Moura et al., 2015; Iglesias-Sarmiento and Deaño, 2016; Raddatz et al., 2017).

#### Domain-General Abilities

Domain-general abilities are part of the overall cognitive functioning and are therefore not strictly limited to mathematics (Geary et al., 2017; Silver et al., 2020). Nevertheless, several studies and multiple meta-analyses have tried to relate certain strengths and weaknesses in domain-general abilities to MD (Henik et al., 2011; Fias et al., 2013; Watson and Gable, 2013). This approach is also supported by fMRI studies. Here, a neuronal network of brain areas linked to domain-specific as well as domain-general abilities was identified when doing arithmetic tasks [for an overview: Kucian (2016), Peters and de Smedt (2018)].

##### Short-Term Working Memory

Most reviews about distinct strengths and weaknesses in domain-general abilities focused on short-term working memory, which compromises of four sub-abilities: auditory and visual-spatial short-term storage, working memory capacity and attentional control (Schneider and McGrew, 2018). Auditory or visual-spatial short-term storage refer to the ability to temporarily store verbal or visual-spatial information, respectively (Lehnert and Zimmer, 2006). Regarding auditory short-term storage, most reviews reported a small weakness in people with MD (Swanson and Jerman, 2006; David, 2012). Only one review by Johnson et al. (2010) found a larger difference between people with and without MD that corresponded to a medium effect size of 0.60. By comparison, effects in visual-spatial short-term storage were in total only analyzed by 2 reviews, which reported both a medium effect size of 0.60 (David, 2012; Peng et al., 2018). In recent years, several studies examined differences between both sub-abilities in people with MD. Since they found larger effects in visual-spatial than in auditory short-term storage (Landerl et al., 2009; Szûcs et al., 2013; Kroesbergen and van Dijk, 2015; Menon, 2016), these studies suggested a general weakness of people with MD in processing visual-spatial information.

Working memory capacity, another sub-abilities of short-term working memory, is the ability to store and process information simultaneously (Swanson, 2012). It is usually measured by tasks in which participants are required to recall a list of items (e.g., letters and digits) in reverse order, or by tasks in which participants have to answer a set of questions while simultaneously memorizing the last word of each question in the given order (i.e., complex span). Regardless of the task, the performance of people with MD was usually lower compared to people without MD and most reviews reported medium effect sizes in working memory capacity. Johnson et al. (2010) estimated a large effect of 0.91 when verbal items had to be memorized while the effect was small for visual items. This difference between type of items was not confirmed by Swanson et al. (2009), who reported medium effect sizes for both type of items. However, since no review reported none or small effect sizes, a general weakness of people with MD in working memory capacity is assumed (Peng and Fuchs, 2014; Attout and Majerus, 2015; Mammarella et al., 2018).

The last sub-ability of short-term working memory is attentional control (or executive functions). Attentional control is understood as the ability to monitor, adapt, and regulate cognitive performance in reaction to changing task settings (van der Sluis et al., 2004). In contrast to the other sub-abilities of short-term working memory, attentional control can be further divided into 3 sub-abilities: Inhibition (i.e., deliberately inhibit a prepotent response), shifting (i.e., shift between tasks), and updating (i.e., update task-relevant information in memory) (Miyake et al., 2000). For people with MD, findings are too ambiguous to draw conclusions yet. Across all sub-abilities, a review by Johnson et al. (2010) reported a small difference between people with and without MD which does not correspond to a distinct weakness. In contrast, Peng et al. (2018) found a large difference between both groups. Peng et al. (2018) also analyzed sub-abilities and estimated a small effect size of 0.37 in inhibition while effect sizes in shifting and updating were 0.75 and 0.76, respectively. No further review about strengths and weaknesses of people with MD in attentional control exists.

##### Other Abilities

Other abilities which were summarized by reviews about MD are processing speed, phonological processing, visual processing and fluid reasoning. However, most of them were not analyzed by more than 2 reviews and findings are mixed. Regarding processing speed, it is the ability to encode information quickly and to perform simple cognitive tasks based on this information fast (Conway et al., 2002). Several sub-abilities can be separated for processing speed (Salthouse, 2000), however, it is mainly perceptual speed which is at its core (Schneider and McGrew, 2018). Perceptual speed basically means to compare simple visual stimuli for differences and similarities very quickly and is measured by visual matching or coding tasks (Ackerman and Beier, 2007). For people with MD, reviews by Johnson et al. (2010) and Peng et al. (2018) reported small and medium effects in favor of people without MD, respectively. However, Johnson et al. (2010) also included studies which measured processing speed by applying rapid naming tasks. Since these tasks require participants to rapidly retrieve the names of well-known stimuli (e.g., letters and numbers) from memory, they are actually measuring retrieval fluency (Koponen et al., 2020). As a consequence, only the review by Peng et al. (2018) remains relevant, which reported a weakness for people with MD in processing speed.

Retrieval fluency, on the other hand, was analyzed by 3 reviews. While Swanson and Jerman (2006) and Peng et al. (2018) reported a weakness corresponding to a medium effect size, Swanson et al. (2009) on the other hand only estimated a small effect size of 0.39 in a follow-up review of his previous one. Retrieval fluency itself is considered as a sub-ability of phonological processing which is described as the ability to “use phonological information (…) in processing written and oral language” (Wagner and Torgesen, 1987). Another sub-ability of phonological processing is phonetic coding (or phonological awareness) in which tasks phonemes, syllables, or onset-rimes have to be manipulated (Treiman and Zukowski, 1996). Only a review by Peng et al. (2018) summarized differences between people with and without MD and reported a distinct weakness in people with MD with a large effect size of 1.31. Since phonological processing is strongly related to writing and especially reading (Wimmer et al., 1991), the weakness found by Peng et al. (2018) can also be compared with reviews analyzing the overall reading and writing ability in people with MD. However, two reviews by Swanson and Jerman (2006) and Swanson et al. (2009) only partially confirm this weakness in phonological processing. While both reviews included the same tasks to measure phonological processing, Swanson and Jerman (2006) reported a small effect size of 0.3 while Swanson et al. (2009) found a large effect size of 1.03.

Besides phonological processing, there is also visual processing, which is the overall ability to perceive, discriminate, manipulate and recall non-linguistic images (Schneider and McGrew, 2018). It is distinct from perceptual speed in a way that it’s not about the fast encoding of simple visual stimuli but the accurate processing of complex visual or visuospatial stimuli. A vast array of sub-abilities have been described in the past (for an overview: Hegarty and Waller, 2005). For people with MD, Peng et al. (2018) reported a small effect size in mental rotation, visuospatial perception and spatial visualization. However, the overall number of studies examining differences in people with and without MD is small and no conclusions regarding strengths and weaknesses in MD can be drawn yet.

The last ability which is commonly summarized by reviews is fluid reasoning. Fluid reasoning is a special case since it is considered as a first-order factor of intelligence on which all other cognitive abilities discussed so far load on as second-order factors (Schneider and McGrew, 2018). Findings by reviews regarding differences between people with and without MD in fluid reasoning are very mixed. They vary from small differences for visual items (Swanson and Jerman, 2006), to medium and large differences for verbal items (Swanson et al., 2009), to large differences regardless of item type (Johnson et al., 2010). Reported differences are in favor of those without MD but given the range of differences it is unclear whether MD is associated with a distinct weakness in fluid reasoning.

To sum it up: Based on published reviews and studies, MD is usually accompanied by domain-specific weaknesses in calculation, fact retrieval and math reasoning; and by domain-general weaknesses in working memory capacity and most likely in visual-spatial short-term storage. For auditory short-term storage the difference between people with and without MD is too small to be considered as a weakness. For all the other domain-specific and domain-general abilities, findings are too ambiguous to draw conclusions.

### Contributions of Present Meta-Analysis Over and Above Existing Ones

Despite the existing evidence given by published meta-analyses, the cognitive profile of MD remains incomplete and not applicable in typical settings. Specifically, we identified three key issues which our meta-analysis addresses. First, DSM or ICD are mostly used to diagnose MD. However, no review so far has applied inclusion criteria similar to diagnostic criteria stated by DSM and ICD. Second, domain-specific abilities have never been systematically summarized yet although they are most important for MD identification. Instead, domain-general abilities were the focus of meta-analyses in the past. And third, it is unclear if strengths or weaknesses in abilities are similar if different scoring types are used (e.g., accuracy and response time). So far, meta-analyses focused mainly on accuracy scores and it is unclear, if response time scores can be used for MD identification.

#### Inclusion Criteria According to DSM and ICD

Regarding the first issue, DSM and ICD define clear criteria to identify MD. That is, MD manifests during the first years of formal schooling and is therefore mostly diagnosed within this period. Overall math performance is below average for age. To test for low math performance, clinical interviews and standardized math achievement tests are used. For tests, performance should be at least 1 standard deviation below the population mean (i.e., percentile rank ≤ 16). Low math performance is not attributable to other causes [e.g., intellectual disabilities, neurological disorders, attention deficit hyperactivity disorder (ADHD)]. Also, to diagnose isolated MD, other learning disabilities, especially reading disabilities (RD), need to be excluded based on the same criteria stated above.

So far, no review applied these inclusion criteria. Regarding the first criterion, onset and age range, only Schwenk et al. (2017) defined an acceptable age range of 6–14 years of age. Johnson et al. (2010) and Peng and Fuchs (2014) included studies with adolescents and applied an upper age limit of 18 and 20, respectively. In reviews by Swanson et al. (2009); David (2012), and Peng et al. (2018) the age range is unclear. For the second criterion, cut-off value, no review is an accordance with the DSM or ICD. The most conservative criterion is used by Schwenk et al. (2017) who only included studies which applied a cut-off of percentile rank 25 or lower. In comparison, Johnson et al. (2010); Peng and Fuchs (2014) and Peng et al. (2018) used a liberal cut-off of percentile rank 35 while reviews by Swanson and Jerman (2006); Swanson et al. (2009), and David (2012) do not mention any cut-off or accepted teacher ratings as criteria instead. For the last criterion, other reasons and comorbidities, only Peng and Fuchs (2014); Schwenk et al. (2017), and Peng et al. (2018) used an IQ criterion of at least 80. However, studies testing students in educational settings do not always apply an additional IQ criterion since an IQ at or greater than 70 is often required to attend regular schools in most countries. Regarding ADHD, it is hardly excluded in studies about MD and therefore not applied as an exclusion criterion in any review about MD either. RD was excluded in multiple reviews (Swanson and Jerman, 2006; Swanson et al., 2009; David, 2012; Peng and Fuchs, 2014; Peng et al., 2018). However, it is often unclear if the inclusion criteria for RD were analogous to those for MD.

That means, no review was completely in accordance with DSM and ICD and it remains unclear if the published strengths and weaknesses in domain-specific and domain-general abilities can be used for MD identification. In particular, strict or liberal cut-offs seem to lead to different cognitive profiles (Murphy et al., 2007; Swanson et al., 2018; Busch et al., 2019). Especially weaknesses in number sense and visual-spatial short-term storage were more pronounced when stricter cut-offs were applied. Also, 20–40% of people with MD also suffer from RD (Moll et al., 2019). Several studies have shown that RD is accompanied by distinctive domain-general weaknesses in phonological processing (Cirino et al., 2015), auditory short-term storage (Mähler and Schuchardt, 2016) and retrieval fluency (Willcutt et al., 2013), which are also present in comorbid cases of MD and RD. Therefore, a proper exclusion of RD is necessary to define a cognitive profile of MD.

#### Summary of Domain-Specific Abilities

Regarding the second issue, prior studies have shown various strengths and weaknesses in domain-specific abilities. While weaknesses in mathematical core abilities like calculation or fact retrieval are mandatory for MD, findings regarding math reasoning and number sense are mixed. Especially number sense can be measured in various ways and consists of different sub-abilities. If those sub-abilities differ in their effect sizes, like they do in number sense, and if no differentiation is made between sub-abilities, conclusions about the overall ability can be seriously biased and can lead to an incorrect MD identification. In addition, number sense is described as a precursor ability to later math abilities like computation (Butterworth et al., 2011; Träff et al., 2020). That means, its development must be considered when MD is identified during the first years of formal schooling. It cannot be ruled out that basic number sense sub-abilities are already developed, even in people with MD, so that no differences between people with and without MD can be found. For example, Fazio et al. (2014) and Schneider et al. (2017) reported low correlations between quantity processing and math performance. Similarly, Schwenk et al. (2017) reported larger differences between people with and without MD in number processing, which is more advanced, than in quantity processing. Since no meta-analysis has systematically summarized number sense sub-abilities yet, the average performance of people with MD during the first years of formal schooling is unknown.

#### Differentiation Between Type of Scoring

Regarding the third issue, abilities can be measured by accuracy or response time scores. So far, only a review by Schwenk et al. (2017) explicitly specified type of scores and summarized scores solely based on response time. For all other reviews it is unclear, what type of scores were included which in turn can seriously affect the size of the reported effects. While most abilities are, per definition, clearly based upon certain type of scores (e.g., processing speed), it is not so obvious for others (e.g., fact retrieval and attentional control). From a statistical perspective, response time scores are usually not normally distributed like accuracy scores. Instead, they follow an asymmetric ex-Gaussian distribution which affects the robustness of most statistical tests (Marmolejo-Ramos et al., 2015). To overcome this problem, data based on response time scores is sometimes transformed in such a way that it is more normally distributed (Lachaud and Renaud, 2011; Speelman and McGann, 2013). However, those procedures vary between studies and skewed distributions often remain skewed even after transformation (Lo and Andrews, 2015). Therefore, mixing accuracy and response time scores also means to mix two different types of data with unpredictable consequences for the overall effect. Thus, it is better to separate between accuracy and response time scores and to control for different data distributions between both scores while simultaneously assume rather similar distributions within. In addition, tasks measuring accuracy or response time scores can also include additional time constraints when there is a time limit or participants are required to work “as fast as possible.” Several studies have reported slower processing speed in people with MD (Proctor, 2012; Niileksela and Reynolds, 2014). For those with MD, additional time constraints can complicate tasks thus negatively affecting their performance compared to people without MD. However, studies about MD rarely analyze response time scores (e.g., Hanich et al., 2001; Mammarella et al., 2013). Consequently, it is unclear if and how response time scores can be used for MD identification and whether the effect sizes are similar regardless of scoring type.

## Aims

As described, people with MD show various strengths and weaknesses in domain-specific as well as domain-general abilities. However, the cognitive profile of MD is still incomplete and previous meta-analysis failed to apply stringent inclusion criteria. The aim of this study was to summarize the domain-specific and domain-general strengths and weaknesses in MD by using diagnostic procedures in accordance with the DSM and ICD. A systematic review and meta-analysis was performed, including studies which compared people with and without MD on several levels of abilities and scoring types.

## Method

### Search Strategies

We searched for studies which compared a group with MD with a typically developing group (TD) on any cognitive measure. The literature search was conducted in February 2021 using the following databases: PsycINFO, MEDLINE, ERIC, ProQuest, PSYNDEX and MathEduc. ProQuest and PSYCINFO were also used to find relevant dissertations and master’s theses. In addition, we searched the following published reviews about MD for relevant citations: Geary (2004, 2010, 2011), Swanson and Jerman (2006), Swanson et al. (2009), Johnson et al. (2010), Raghubar et al. (2010), David (2012), Peng and Fuchs (2014), Shin and Bryant (2015), Peng et al. (2016), Peng et al. (2018), and Vanbinst and de Smedt (2016).

Depending on the database, we searched with English and/or German search terms using title, abstract and keywords as search fields. To find studies comparing people with and without MD, we combined keywords for *MD* with keywords for *difference* and *group membership* using AND (see Supplementary Material).

**FIGURE 1:**
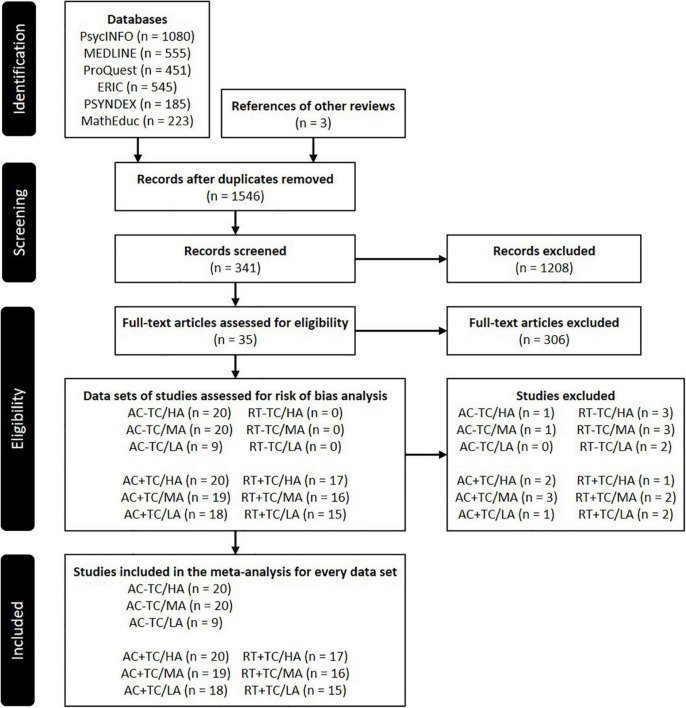
Flow chart of literature search. AC, accuracy; RT, response time; TC, time constraints; HA, high-level ability; MA, medium-level ability; LA, low-level ability.

Figure 1 provides a flow chart of the literature search according to Moher et al. (2009). The literature search initially provided 3039 studies. After removal of duplicates and application of every inclusion criteria, the number of studies was narrowed down to 35. The quality of those studies was assessed by using the *Appraisal Tool For Cross-Sectional Studies* (*AXIS*) (Downes et al., 2016), which confirmed proper study quality for all studies. Three studies were based on the same data set (de Weerdt et al., 2013a,b; Desoete and de Weerdt, 2013), which we therefore treated as a single study. One study (Schleifer and Landerl, 2011) consisted of three sub-studies with different samples, which we considered as 3 different studies. The remaining total pool of 34 studies was divided into 12 different data sets based on scoring and level of every outcome (see Section “Coding Procedures”). For every data set we applied risk of bias analysis and excluded outcomes with a very small number of studies (see Section “Statistical Methods”). Depending on the data set, 1–4 studies were excluded. For data set RT - TC (i.e., response time scores without time constraints) only 3 studies could be identified in total. After risk of bias analysis, no study remained, thus we could not perform any meta-analysis for data set RT - TC.

### Inclusion Criteria

The MD and TD groups each had at least two persons. All included studies were published either in English or German. Because of the language skills of both authors, no other languages could be considered. Publication year of all studies was not before 1992 (publication year of ICD-10).

The MD was defined as a percentile rank at or below 16 in a standardized math test, a lag of at least 18 chronological or 15 instructional months in math (i.e., at least 1.5 years or 1.5 grades), or an existing diagnosis of MD (DSM- IV/5: 315.1; ICD-10: F81.2). Although other criteria and cut-offs exist to diagnose MD (Möller et al., 2012; Kaufmann et al., 2013), we chose PR ≤ 16 (i.e., one standard deviation) since it is recommended as cut-off in DSM-5.

All participants of our included studies received regular education (i.e., no special education) and were between 8 and 12 years old. In case years of age were not reported, participants had to attend 2nd to 6th grade. According to DSM and ICD, MD manifests during the first years of formal schooling and is mostly diagnosed during primary education (Nelson and Powell, 2018). Although very severe math difficulties can already be identified at a very young age (Stock et al., 2010), math performance of students in 1st grade still varies too much for MD identification (Kohn et al., 2013; Koponen et al., 2018). For this reason, we restricted the age range to the typical age range in which MD is usually identified.

For every study, MD and TD groups were matched for age or grade and for gender to control for age-moderated and gender-moderated differences between both groups, respectively.

To control for other causes for low math performance, only studies about MD were included in which, first, math performance was not primarily associated with a genetic or neurological disorder like fragile X syndrome (Murphy, 2009), Turner syndrome (Baker and Reiss, 2016), 22q11.2 deletion syndrome (de Smedt et al., 2009; Brankaer et al., 2016), neurofibromatosis type I (Orraca-Castillo et al., 2014), cerebral palsy (van Rooijen et al., 2015), epilepsy (van Iterson et al., 2015). Second, MD was not primarily associated with low birth weight or premature birth (Taylor et al., 2009; Jaekel and Wolke, 2014). And third, studies controlled for intellectual disability which was defined in accordance with DSM and ICD as an IQ lower than 70. Since an IQ of 70 is relatively low, studies reporting an “average IQ” as their selection criteria were included too.

Because 20–40% of people with MD also show low reading performance (Dirks et al., 2008; Landerl and Moll, 2010; Fischbach et al., 2013; Willcutt et al., 2013; Moll et al., 2014), we included only studies which controlled for reading difficulties (RD). Analogous to MD, RD was defined as a percentile rank above 16 in a standardized reading test, a lag of less than 18 chronological or 15 instructional months in reading (i.e., less than 1.5 years or 1.5 grades), or no existing diagnosis of RD (DSM-IV/5: 315.0; ICD-10: F81.0; ICD-11: 6A03.1).

As a final inclusion criterion, all studies had to report enough data to compute effect sizes based on the standardized mean difference between both groups.

### Coding Procedures

For the descriptive variables, we coded study, sample, and diagnostic characteristics. Study characteristics included (a) year of publication and (b) type of publication. Sample characteristics of MD and TD group were (a) sample size, (b) mean age in months, and (c) percentage of male participants. Diagnostic characteristics contained (a) measures of IQ, reading and math performance; (b) criteria used to exclude intellectual disabilities and RD, and criteria used to diagnose MD; and (c) if ADHD was excluded or not.

For the outcome variables, we used an exploratory approach according to Brown et al. (2003). That meant instead of defining a coding scheme for study outcomes in advance we derived relevant coding categories for the outcomes from the included studies themselves. This approach reduced the risk of excluding important data *a priori* by not-coding relevant outcomes and was therefore most suitable to derive a comprehensive cognitive profile of MD. Furthermore, precision of effect size estimations increased since we could consider dependent correlations between outcomes which could have otherwise been ignored (Riley, 2009).

We started this exploratory coding approach by separating between the *scoring* and *level* of every outcome of every study. For scoring, we coded for each outcome if *accuracy* (*AC*; e.g., number of solved items) or *response time* (*RT*; e.g., mean response time for solved items) was measured and if any *time constraints* were involved (*TC*; e.g., time limit, instructed to “work as quickly as possible”). This resulted in 4 different categories for scoring: Accuracy without time constraints (AC − TC) and with time constraints (AC + TC) as well as response time without time constraints (RC − TC) and with time constraints (RT + TC).

For level, we coded every outcome on the following three hierarchical levels of abilities in accordance to their description in the Introduction: *High-level ability* (*HA*), *medium-level ability* (*MA*), and *low-level ability* (*LA*). First, we assigned study outcomes to the same low-level abilities when they shared a similar operationalization (e.g., counting forward from 1 to 10 and counting backward from 8 to 2 was defined as low-level ability number word knowledge). Second, we assigned similar low-level abilities to its respective medium-level abilities (e.g., low-level abilities number word knowledge and quantity processing were assigned to medium-level ability number sense). Third, we assigned medium-level abilities to its corresponding high-level abilities (e.g., medium-level abilities number sense and computing were assigned to high-level ability mathematics). For better understanding, high-level, medium-level and low-level ability can also be referred to as first-order, second-order and third-order factor, respectively. If we could not assign a study outcome to a single low-level ability (e.g., index scores which comprised several low-level abilities) this outcome was only assigned to its respective medium-level or high-level ability (e.g., a single outcome comprising tasks measuring quantity processing and numerical relations was only assigned to medium-level ability number sense and high-level ability mathematics). Taken together, a single outcome was coded on one of four different categories for scoring (AC − TC, RT − TC, AC + TC, and RT + TC) and on three different levels (HA, MA, and LA) which resulted in 4 × 3 independent data sets of coded outcomes. See Supplementary Table 1 for the final coding scheme.

Based upon the set of included studies the first author and a student assistant developed the coding scheme by scanning all reported study outcomes as described above. In case of disagreements, the relevant study outcomes were discussed until a consensus was reached. After the coding scheme was finalized, all descriptive and outcome variables were coded. The first 10 studies were double-coded by the first author and the student assistant. Inter-rater agreement varied between 88 and 97% using Cohen’s kappa (Cohen, 1960) for nominal and intraclass correlation coefficients (Hallgren, 2012) for ordinal or higher levels of measurement, respectively. Lower agreement rates were due to incomplete descriptions of diagnostic cut-off criteria, tests, and measures provided by the studies.

In case of missing data, we contacted the study’s authors. If they didn’t provide the data, effect sizes and standard deviations were computed by transforming reported data using formulas provided by Borenstein (2009). If transforming was not possible, we differentiated between descriptive and outcome data. In case of missing descriptive data, studies were excluded from further analysis if the missing data was related to the inclusion criteria of the meta-analysis. This was done to ensure a clean data set strictly according to our criteria. For missing outcome data, studies were excluded if there was a high risk of a selective outcome reporting bias (Pigott et al., 2013) according to AXIS. Serious selective outcome reporting indicated that a study was strongly biased toward certain conclusions. This also made it unclear, if the methods (esp. statistical procedures) were properly conducted and if we could trust the outcomes which were reported. For this reason, we excluded those studies instead of trying to address this issue by using risk of bias analysis. However, no study had to be excluded because of serious selective outcome reporting.

We assessed the quality of each study by using the *Appraisal Tool For Cross-Sectional Studies* (*AXIS*) (Downes et al., 2016) which contains 20 yes/no questions evaluating the quality of reporting, study design, and possibility of bias of cross-sectional studies (e.g., “Was the target population clearly defined?”, Were the limitations of the study discussed?”). The final judgment about study quality is determined by an overall subjective assessment of every item since no numerical rating scale is provided.

Initially we coded 9 high-level abilities, 19 medium-level abilities and 13 low-level abilities. After we separated the overall data set into the corresponding data sets for accuracy and response time with or without time constraints and after we excluded outcomes with a very small number of studies only 5 high-level abilities, 9 medium-level abilities and 8 low-level abilities remained which were included in the meta-analysis. For domain-specific abilities most outcomes fulfilled our criterion and were reported by at least 3 studies with at least 2 different first authors (see Section “Statistical Methods”). However, for domain-general abilities we had to exclude most of the coded outcomes. We did not find more than 2 studies for high-level abilities processing speed, visual processing, learning efficiency, and comprehension knowledge. As a result, our analysis about domain-general abilities only covered short-term working memory (16 studies), reaction and decision speed (3 studies) and fluid reasoning (3 studies).

### Statistical Methods

All analyses were conducted using R (R Core Team, 2020) and R package robumeta (Fisher et al., 2017). We used the standardized mean difference between the MD and TD group as effect size measure. For this reason Hedges’ *g* (Hedges, 1981) was calculated which corrects Cohen’s *d* (Cohen, 1969) for a small sample bias.

Before conducting the meta-analysis, we applied several measures to reduce the risk of bias in individual studies (e.g., selective reporting) and across studies (e.g., publication bias). Studies were excluded if AXIS indicated a low study quality and a high risk of reporting bias. To reduce the influence of outliers within each data set we applied a 90% Winsorizing to these effect sizes (Dixon, 1960). That is, effect sizes below the 5th or above the 95th percentile were set to the 5th or 95th percentile, respectively.

Outcomes were excluded, if they were not reported by at least 3 studies which in addition were published by at least 2 different first authors. The validity of a meta-analysis depends more on the expected heterogeneity and quality of the studies included than the total number of included studies (Valentine et al., 2010). Since our study sample was well defined because of stringent inclusion criteria (e.g., small age range and clinical diagnostic criteria) and since each study was critically appraised by using a checklist, we expected rather similar effect sizes and smaller standard errors for outcomes. For this reason, we accepted a smaller number of studies per outcome as sufficient. We specifically settled on 3 studies as the minimum number of studies per outcome in the unlikely event that the effect sizes of 2 studies were very contrasting so that the effect size of the third study could give us the right direction of the overall average effect size. The additional criterion of at least 2 different first authors for each outcome was necessary to reduce the risk of authorship bias (Moulin and Amaral, 2020) since the number of researchers focusing on MD is small.

Regarding the analysis, most studies provided more than one outcome and multiple effect sizes for each outcome. To account for statistical dependencies of multiple outcomes and effect sizes from the same studies (Jackson et al., 2011; Moeyaert et al., 2017) we performed a robust variance estimation method based on a random effects model (RVE; Raudenbush, 2009; Hedges et al., 2010; Tipton, 2015). Although we used strict inclusion criteria, we applied a random effects model because we still expected effects of the same outcomes to vary across studies. The RVE method corrects the standard error of the average effect size estimate by taking into account the correlations between effect sizes from the same sample (i.e., same study). For this, an estimation of the mean correlation between all pairs of effect sizes within a study is needed. We chose *p* = 0.70 which is a rather conservative estimate to reduce the risk of a type I error. In addition, we performed sensitivity analysis with various estimates of *p* to examine the robustness of our results. But, as Hedges et al. (2010) already pointed out, the estimate of the mean correlation has actually no considerable effect on the standard error estimates.

After the analysis, we conducted several meta-regressions to assess risk of bias across studies. For every outcome we applied the same criterion regarding minimum number of studies as for the overall analysis. That is, a meta-regression was only performed if the respective moderator was reported by at least 3 studies which were published by at least 2 different first authors. To examine publication bias, we applied Egger’s regression test (Egger et al., 1997) and Funnel plot tests (Sterne and Egger, 2001) which examine the influence of the standard errors and the sample size on the estimated average effect sizes, respectively. Both tests, when significant, indicate the presence of a small-study effect which means that too many studies with small sample sizes and large effect sizes are in the data set. However, it should be noted that both tests are prone to false positive results, especially when heterogeneity in the data is high (Rodgers and Pustejovsky, 2020). We created no funnel or forest plots since both type of plots did not account for statistically dependent effect sizes thus making any interpretation misleading (Doleman et al., 2020). To control for biases based on the characteristics of the TD and MD group (e.g., selection bias) we analyzed the moderating effect of severity of MD (i.e., difference in math performance between groups) as well as differences in IQ and reading performance between groups.

## Results

### Study Characteristics

In total and across all data sets 34 studies were included, comprising 320 effect sizes. The total MD sample consisted of 680 unique people (43.6% male) with a mean age of 117.3 months and the total TD sample covered 1565 unique people (48.4% male) with a mean age of 117.5 months. Regarding IQ, 85.3% of all studies measured non-verbal IQ or a combination of non-verbal and verbal IQ. Cut-off used to exclude low IQ ranged from 80 to 90. Regarding reading performance, 88% of all studies measured reading fluency or a combination of reading fluency and reading comprehension. Most studies (85.3%) applied percentile ranks to exclude RD, which ranged from percentile rank 16 (the pre-defined minimum) to 40. Only 5.9% or 8.8% of all studies used delays in months or an existing diagnosis of RD as exclusion criteria, respectively. Regarding math performance, 97.1% of all studies measured computation or a combination of computation with number sense and/or math reasoning. Similar to reading performance, most studies applied percentile ranks to define MD, which ranged from percentile rank 5–16 (the pre-defined maximum). For MD, 20.6% or 8.8% of all studies used delays in months or an existing diagnosis of MD as inclusion criteria, respectively. Regarding ADHD, less than half of all included studies (47.1%) controlled for ADHD within their samples. Descriptive information and assignment of studies to each data set are listed in Table 1.

**TABLE 1:** Descriptive characteristics of included studies and data sets.

Study	ID^a^	Type	MD			TD			IQ		Reading		Math		ADHD Excl.
			*n*	*M*_*age*_	Male	*n*	*M*_*age*_	Male	Meas.	Cut-off^b^	Meas.	Cut-off^c^	Meas.	Cut-off^c^	
Ashkenazi et al., 2009	1	A	16	113.0	31.3	13	113.0	15.4	NV	90	RF/RC	25	NS/CO/MR	≥20 i.m.	Yes
Ashkenazi et al., 2013	2	A	11	113.8	18.2	11	114.1	18.2	NV	90	RF/RC	25	NS/CO/MR	≥20 i.m.	Yes
Censabella and Noël, 2008	3	A	20	124.0	50.0	20	125.9	50.0	NV/V	85	RF/RC	30	CO	≥20 i.m.	No
Compton et al., 2012	4	A	537		50.3	32		52.3	NV/V	80	RF/RC	40	NS/CO	16	No
de Weerdt et al., 2013a	5	A	45	120.9	42.2	22	117.6	27.3	NV/V	80	RF	25	NS/CO	11	Yes
de Weerdt et al., 2013b	5	A	45	120.9	42.2	22	117.6	27.3	NV/V	80	RF	25	NS/CO	11	Yes
Desoete and de Weerdt, 2013	5	A	45	120.9	42.2	22	117.6	27.3	NV/V	80	RF	25	NS/CO	11	Yes
Evans, 2008	6	D/M	72	116.6	40.3	65	122.5	47.7	NV/V	90	RF	16	CO	16	No
Heine et al., 2013	7	A	20	98.4	45.0	20	99.2	50.0	NV/V	85		21	NS/CO	5	Yes
Karakonstantaki et al., 2018	8	A	31	117.5	64.5	19	117.2	52.6	NV/V	80	RF	25	NS/CO	16	Yes
Kaufmann et al., 2003	9	A	18	112.8	50.0	6	115.2	66.7		Avg.		ICD		ICD	No
Kucian, 2005	10	D/M	10	110.4	50.0	9	121.2	44.4		Avg.	RF	ICD	NS/CO	ICD	Yes
Landerl et al., 2009	11	A	42	109.5		20	110.4		NV/V	85	RF/RC	25	NS/CO	16	Yes
Mähler and Schuchardt, 2016	12	A	31	107.1	48.4	18	102.5	27.8	NV/V	80	RF	16	NS/CO/MR	16	Yes
Mammarella et al., 2018	13	A	24	116.8	41.7	24	117.4	58.3	NV/V	Avg.	RF	16	NS/CO	16	No
Mejias et al., 2012	14	A	23	118.1	60.9	23	118.4	39.1	NV/V	80	RF	16	CO/MR	16	No
Miles and Stelmack, 1994	15	A	10	140.4	100.0	8	129.6	100.0	NV/V	90	RF	16	NS/CO	16	No
Moll et al., 2015	16	A	32	107.5	47.0	17	112.8	35.0	NV/V	Avg.	RF	16	NS/CO	16	No
Morsanyi et al., 2013	17	A	16	125.0	43.8	13	123.0	46.2	NV/V	80	RF/RC	16	NS/CO/MR	16	No
Morsanyi et al., 2018	18	A	20	117.7	50.0	20	113.8	65.0	NV/V	85	RF/RC	16	NS/CO/MR	16	No
Mussolin et al., 2010a	19	A	15	131.5	60.0	15	126.1	46.7	NV/V	85	RF/RC	16	CO/MR	≥24 c.m.	Yes
Mussolin et al., 2010b	20	A	15	123.7	46.7	15	121.8	60.0	NV/V	85	RF/RC	16	CO	16	No
Peng et al., 2012	21	A	30	131.7	43.3	18	132.1	38.9	NV	Avg.	RF	16	NS/CO/MR	16	No
Raddatz et al., 2017	22	A	40	111.6	22.5	20	112.6	30.0	NV	85	RC	16	NS/CO	7	Yes
Reikerås, 2006	23	A	205		46.3	28		64.3		Avg.	RF/RC	20	NS/CO	16	No
Rotem and Henik, 2013	24	A	24	142.8	50.0	16	146.4	43.8	NV	90	RF/RC	35	NS/CO/MR	≥24 i.m.	Yes
Rousselle and Noël, 2008	25	A	22	105.3	36.4	18	102.7	33.3	NV/V	85	RF/RC	16	NS/CO	16	No
Schleifer and Landerl, 2011 (Study 1)	26	A	12	99.3	50.0	12	99.0	50.0	NV/V	85	RF	16	NS/CO	7	Yes
Schleifer and Landerl, 2011 (Study 2)	27	A	19	111.0	52.6	19	111.1	26.3	NV/V	85	RF	16	NS/CO	7	Yes
Schleifer and Landerl, 2011 (Study 3)	28	A	21	121.3	57.1	21	125.3	47.6	NV/V	85	RF	16	NS/CO	7	Yes
Schuchardt et al., 2008	29	A	30	108.8	50.0	17	103.4	29.4	NV/V	80	RF	16	NS/CO/MR	16	No
Schuchardt and Mähler, 2010	30	A	30	108.0	50.0	22	102.0	27.3	NV/V	80	RF	ICD	NS/CO/MR	ICD	No
van der Sluis et al., 2004	31	A	19	127.4	47.4	18	128.7	27.8	V	Avg.	RF	<15 i.m.	CO	≥15 i.m.	No
van der Sluis et al., 2005	32	A	18	127.7	50.0	17	128.3	23.5	V	Avg.	RF	<15 i.m.	CO	≥15 i.m.	No
Wang et al., 2012	33	A	45	132.0	62.2	45	129.0	73.3	NV/V	90	RC	16	NS/CO	16	Yes
Willburger et al., 2008	34	A	42	109.5	40.5	19	110.7	21.1	NV/V	85	RF	25	NS/CO	16	Yes
Separated by data set															
AC − TC/HA	^d^		439	115.4	42.9	1286	115.9	46.7							
AC − TC/MA	^d^		439	115.4	42.9	1286	115.9	46.7							
AC − TC/LA	^e^		215	112.3	46.9	470	112.1	45.2							
AC + TC/HA	^f^		385	118.5	45.5	550	118.3	49.0							
AC + TC/MA	^g^		377	117.9	42.4	540	117.2	46.2							
AC + TC/LA	^h^		359	117.1	42.6	510	116.4	46.3							
RT + TC/HA	^i^		320	119.7	43.0	403	119.1	47.5							
RT + TC/MA	^j^		300	120.1	41.7	383	119.2	47.3							
RT + TC/LA	^k^		284	118.4	41.5	359	117.6	47.2							
Total			680	117.3	43.6	1565	117.5	48.4							

*Meas., measure used to assess IQ, reading, and math performance; cut-off, cut-off used to exclude people with intellectual disability and RD, or to include people with MD; ADHD excl., if study controlled for ADHD; A, journal article; D/M, dissertation or master’s thesis; NV, non-verbal; V, verbal; avg., average; RF, reading fluency (word and non-word); RC, reading comprehension; NS, number sense; CO, computation; MR, math reasoning; c.m, chronological months; i.m, instructional months; AC, accuracy; RT, response time; TC, time constraints; HA, high-level ability; MA, medium-level ability; LA, low-level ability.*

*^a^Studies with identical ID were treated as a single study.*

*^b^IQ, unless otherwise specified.*

*^c^Percentile rank, unless otherwise specified.*

*^d^Included IDs: 3, 4, 5, 6, 7, 9, 11, 12, 13, 15, 16, 17, 20, 21, 22, 24, 28, 29, 31, 34.*

*^e^Included IDs: 6, 7, 9, 15, 17, 21, 22, 24, 28.*

*^f^Included IDs: 2, 3, 5, 6, 7, 8, 9, 10, 13, 14, 15, 17, 18, 19, 20, 21, 24, 28, 33, 34.*

*^g^Included IDs: 2, 3, 5, 6, 7, 8, 9, 10, 13, 15, 17, 18, 19, 20, 21, 24, 28, 33, 34.*

*^h^Included IDs: 2, 3, 5, 6, 7, 8, 9, 10, 13, 15, 17, 18, 19, 21, 24, 28, 33, 34.*

*^i^Included IDs: 1, 3, 5, 8, 10, 15, 17, 18, 19, 20, 21, 23, 25, 26, 27, 30, 32.*

*^j^Included IDs: 1, 3, 5, 8, 10, 15, 18, 19, 20, 21, 23, 25, 26, 27, 30, 32.*

*^k^Included IDs: 1, 3, 5, 8, 10, 15, 18, 19, 20, 21, 25, 26, 27, 30, 32.*

### Estimated Effects for High-Level Abilities, Medium-Level Abilities, and Low-Level Abilities

The size of the effect is interpreted according to Cohen (1988) (i.e., none or not relevant: *g* < 0.2; small: 0.2 ≤ *g* < 0.5; medium: 0.5 ≤ *g* < 0.8; large: *g* ≥ 0.8). In addition, only medium to large effect sizes were considered as a particular strength or weakness. Positive effect sizes reflect better scores in favor of the TD group (e.g., higher accuracy and faster response time) and vice versa. We had to control for type I error when determining statistical significance since degrees of freedom were small for some abilities. In line with Tanner-Smith et al. (2016), *p* < 0.05 was used when degrees of freedom were greater than or equal 4 and *p* < 0.01 when degrees of freedom were less than 4.

**FIGURE 2:**
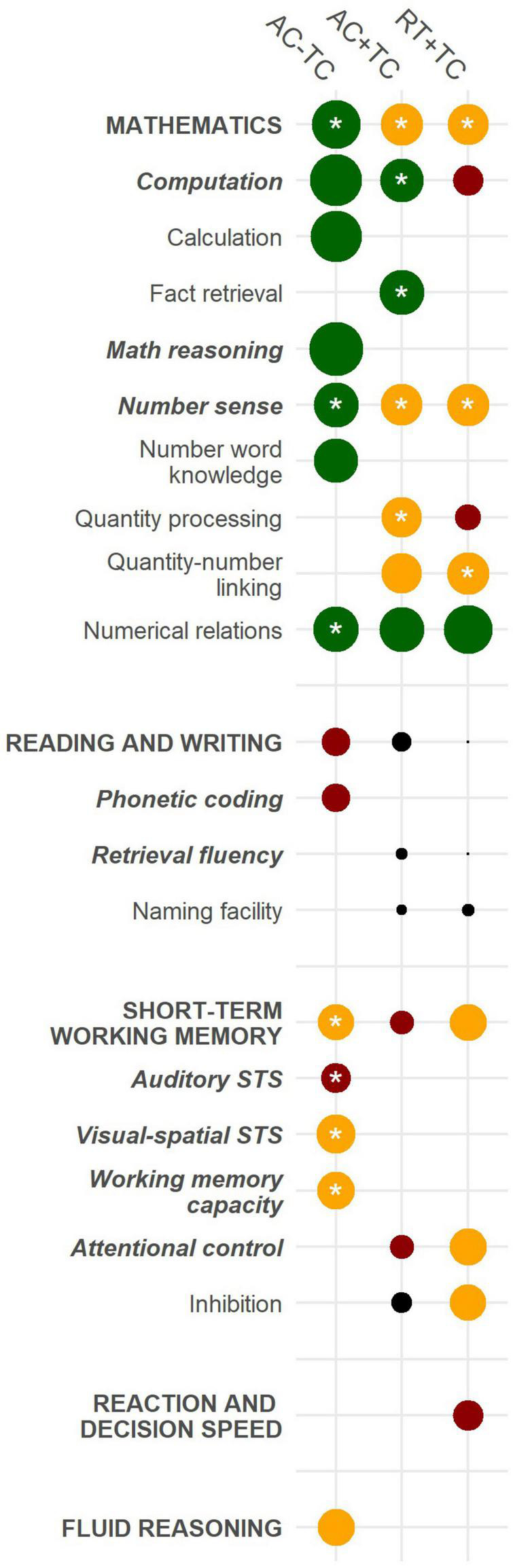
Estimated effects for domain-specific and domain-general abilities. AC, accuracy; RT, response time; TC, time constraints; STS, short-term memory; font formatting reflects ability level (high-level ability, bold and uppercase; medium-level ability, bold and italic; low-level ability, no formatting); size of circle reflects size of effect (i.e., the larger the circle the larger the effect in favor of TD); color of circle reflects type of effect (green = large, yellow = medium, red = small, black = none); *significant effect sizes.

Table 2 lists the estimated effects for high-level abilities, medium-level abilities, and low-level abilities for data sets AC − TC and AC + TC, and RT + RT. To facilitate comparison of scoring and level of outcomes, not all statistics have been reported in Table 2 (e.g., confidence intervals). An extensive list of all statistics is provided by Supplementary Table 2. For a better overview of the results, the estimated effects are shown in Figure 2.

**TABLE 2:** Results of random-effects model with RVE for data sets AC − TC, AC + TC, and RT + TC.

Outcome (HA/MA/LA)^a^	Accuracy no time constraints					Accuracy with time constraints					Response time with time constraints				
	*ST*	*ES*	*df*	*g*	*SE*	ST	ES	*df*	*g*	*SE*	ST	ES	*df*	*g*	*SE*
Mathematics	9	45	7.05	1.04***	0.17	16	72	13.26	0.78***	0.12	13	56	10.47	0.71***	0.14
Computation	5	13	2.49	1.19	0.32	8	26	5.55	0.81**	0.19	3	6	1.71	0.38	0.43
Calculation	5	13	2.78	1.15	0.24										
Fact retrieval						7	22	4.53	0.87*	0.22					
Math reasoning	3	3	1.78	1.29	0.11										
Number sense	6	29	4.57	0.87**	0.17	13	46	9.30	0.75***	0.16	12	50	8.95	0.77***	0.15
Number word knowledge	4	8	2.52	0.84	0.20										
Quantity processing						6	25	3.87	0.69**	0.10	4	22	2.23	0.27	0.08
Quantity-number linking						7	9	3.56	0.69	0.33	7	12	4.17	0.77**	0.10
Numerical relations	6	19	4.58	0.9**	0.17	5	12	3.19	0.87	0.28	5	16	3.66	1.03	0.27
Reading and writing	3	3	1.88	0.33	0.04	6	20	3.68	0.14	0.12	3	8	1.94	0	0.15
Phonetic coding	3	3	1.91	0.33	0.04										
Retrieval fluency						5	15	2.65	0.04	0.05	3	8	1.94	0	0.15
Naming facility						5	13	2.63	0.03	0.07	3	8	1.94	0.05	0.18
Short-term working memory	16	83	12.52	0.56***	0.07	5	11	2.83	0.23	0.08	5	16	3.48	0.58	0.15
Auditory short-term storage	9	30	7.16	0.36***	0.07										
Visual-spatial short-term storage	9	24	4.80	0.66**	0.12										
Working memory capacity	13	27	6.89	0.61**	0.11										
Attentional control						5	11	2.84	0.23	0.08	5	16	3.48	0.58	0.15
Inhibition						4	7	2.03	0.16	0.05	5	13	3.23	0.56	0.16
Reaction and decision speed											3	3	1.25	0.38	0.18
Fluid reasoning	3	5	1.31	0.58	0.12										

*Positive effect sizes indicate a higher accuracy or faster response time in the TD group. AC, accuracy; RT, response time; TC, time constraints; HA, high-level ability; MA, medium-level ability; LA, low-level ability; ST, number of studies; ES, number of effect sizes; g, Hedges’ g; SE, standard error.*

*^a^Subordination of outcome reflects content, i.e., high-level ability (no indent), medium-level ability (medium indent), and low-level ability (large indent).*

*p < 0.05 (if df ≥ 4), **p < 0.01 (if df < 4 or df ≥ 4), ***p < 0.001 (if df < 4 or df ≥ 4).

All included outcomes were in favor of the TD group. However, for medium-level abilities and low-level abilities the number of studies was small and *p* < 0.01 was mostly used as significance level. As a consequence, most outcomes were not statistically significant.

Regarding domain-specific abilities, the MD group showed significant weaknesses in high-level ability mathematics regardless of data set. However, weaknesses in accuracy without any time constraints were usually large compared to medium weaknesses in accuracy with time constraints or response time with time constraints. Especially low-level ability calculation and medium-level ability math reasoning were more affected in people with MD compared to low-level ability fact retrieval. For medium-level ability number sense, results were mixed and dependent on the data set. Effect sizes for low-level ability numerical relations (i.e., mostly symbolic comparison tasks) were large in all data sets, but only significant in data set AC - TC. This was interesting compared to low-level ability quantity processing (i.e., mostly non-symbolic comparison tasks). Here, effect sizes were medium and significant in data set AC + TC while only a small and non-significant effect size could be found for data set RT + TC. Also, low-level ability quantity-number linking (e.g., subitizing and dot enumeration) was larger and significant for data set RT + TC compared to data set AC + TC. Low-level ability number word knowledge (e.g., counting) was only reported for data set AC − TC and a large effect size was found.

For high-level ability reading and writing and their corresponding medium and low-level abilities effect sizes were either small or not-relevant and not-significant. Also, for low-level ability naming facility, which included RAN tasks with numbers as stimuli, we did not find a weakness for people with MD. However, small effect sizes for reading and writing were to be expected since we controlled for RD in our sample of studies.

Regarding domain-general abilities, only some effect sizes for high-level ability short-term working memory were significant. In particular, effect sizes for medium-level abilities visual-spatial short-term storage and working memory were medium compared to small effect sizes for medium-level ability auditory short-term storage. We could not find significant effects for medium-level ability attentional control. The only difference was that effect sizes for data set RT + TC were larger compared to data set AC + TC. However, this was moderated more by the tasks than by MD status since tasks measuring attentional control (e.g., stroop task and flanker task) are usually designed around response time differences.

For high-level ability reaction and decision speed, people with MD showed no significant differences compared to people without MD. Also, no significant difference was found for high-level ability fluid reasoning which was also because all studies controlled for low IQ within their samples.

### Risk of Bias Analysis

Results of risk of bias analysis are shown in Supplementary Table 3. For some outcomes the number of studies was too small to perform moderator analysis. To determine statistical significance, we used the same procedure as described above (i.e., *p* < 0.01 for *df* < 4; *p* < 0.05 for *df* ≥ 4). We only identified publication bias when outcomes were measured using time constraints (AC + TC and RT + TC). Egger’s test indicated a publication bias in data set AC + TC and in data set RT + TC for high-level ability mathematics and medium-level ability number sense, respectively. Otherwise, no other moderating effect for any ability could be identified. That is, sample size (i.e., funnel plot test), differences in IQ or reading performance and severity of MD did not significantly moderate the estimated effects in any data set.

## Discussion

This meta-analysis compared people with and without MD regarding their domain-specific and domain-general abilities. Outcomes differed based on their type of scoring (accuracy or response time with/without time constraints) and their level (high-level ability, medium-level ability, and low-level ability). A cognitive profile of MD based on criteria given by DSM and ICD was found, which is characterized by significant weaknesses in the in the following abilities: High-level abilities mathematics (AC − TC, AC + TC, and RT + TC), short-term working memory (AC − TC); medium-level abilities computation (AC + TC), number sense (AC − TC, AC + TC, and RT + TC), visual-spatial short-term storage (AC − TC), working memory capacity (AC − TC); and low-level abilities fact retrieval (AC + TC), quantity processing (AC + TC), quantity-number linking (RT + TC), and numerical relations (AC − TC). Based on the included studies, no particular strength in people with MD compared to those without MD could be found. Severity of MD, group differences in reading performance and IQ did not significantly moderated the results.

### Domain-Specific Abilities: Various Weaknesses Across Outcome Levels and Scoring

Regarding domain-specific abilities, this was the first meta-analysis to even summarize abilities in this area. As expected, MD affects all mathematical abilities which is in line with previous research defining MD as a heterogeneous disorder (Kaufmann et al., 2013; Karagiannakis et al., 2014).

#### Computation and Math Reasoning

For computation, results are mostly consistent with previous studies reporting severe weaknesses in people with MD (Busch et al., 2015). Interestingly, we only found a small effect size for response time scores based on 3 studies. Kucian (2005) is a brain-imaging studies in which 6th grade students had to solve arithmetic facts by choosing between two different answers. Rotem and Henik (2013) applied a similar paradigm wherein arithmetic facts (e.g., 3 × 6 = 18) were presented to 6th grade students and they had to decide if the total equation was right or wrong. The sample in Raddatz et al. (2017) was younger and consisted of students from 2nd to 4th grade which had to solve simple and more complex calculation tasks. Although all 3 studies differ in their study design and sample, these methodological differences do not seem sufficient for us to explain why people with MD answer nearly as fast as people without MD (RT + TC) but simultaneously struggle to find the correct answer when investigating accuracy scores (AC + TC). One way out of this dilemma is to look at how response time scores are usually being analyzed. That is, only response times of correct answer are considered for further analyses, which was also the case in these 3 studies. Applied to our overall review, this means that people with MD are as fast as people without MD in calculation and especially fact retrieval when they know the results. “Knowing the results” basically means, that people with MD can retrieve facts from memory as easily and as fast as people without MD provided they have memorized those facts beforehand. This hypothesis is supported by the fact that we did not find any weakness in retrieval fluency and especially naming facility (i.e., rapid naming), most likely because we excluded reading disabilities in our sample (Moll et al., 2019). Nevertheless, further studies examining response time scores in fact retrieval tasks are needed to provide a final answer. Also, the large effect sizes for calculation was not significant (*p* = 0.022) when using *p* < 0.01. Only 5 studies applied an additional calculation task, after they identified people with or without MD based (partially) on calculation tasks. Since weaknesses in calculation are a core element of MD, we can only strongly assume this effect size to become significant with more studies.

One of the largest effect sizes overall was found for math reasoning. As mentioned earlier, math reasoning can be operationalized in various ways (e.g., word problems and number series) and it is not clear yet what math reasoning is actually about and how it is related to fluid reasoning. Unfortunately, we could not solve this riddle. Only 3 studies could be included in our meta-analysis, which did not allow for any further differentiation of sub-abilities. Also, all 3 studies did not report IQ scores and hence no meta-regression was possible to check how math reasoning is related to fluid reasoning.

#### Number Sense

Regarding number sense, this meta-analysis clearly underlines the essential role of number sense for MD in general (Butterworth et al., 2011) while also gives important hints regarding sub-abilities and scoring. Most interesting, people with MD showed large weaknesses in numerical relations regardless of scoring while weaknesses in quantity processing and quantity-number linking were smaller and inconsistent across types of scoring. In development models about mathematics, at first children learn to process non-symbolic quantities (e.g., •⁣•⁣• > •) and to link non-symbolic quantities to respective symbolic quantities (i.e., •⁣•⁣• = 3 = “three”). The subsequent understanding of relations between those numbers (e.g., 6 > 4) is seen as the final step to process numbers correctly (Träff et al., 2020). While there is an ongoing debate on how representations of non-symbolic and symbolic quantities are connected to one another, quantity processing undeniably plays an important part in early math development (Kuhn et al., 2016). Nevertheless, its correlation with later math abilities has proven to be rather low (Fazio et al., 2014). Considering that our sample consisted of 2nd to 6th grade students, we assume that our participants with and without MD were already too matured in their overall math development (McCaskey et al., 2018) so that weaknesses in more basic number sense abilities like quantity processing and quantity-number linking were less prevalent. From an empirical perspective, our results are in line with several studies showing that weaknesses in numerical relations are more severe and robust in people with MD than weaknesses in other number sense abilities (de Smedt et al., 2013; Sasanguie et al., 2013; Vanbinst and de Smedt, 2016; Schneider et al., 2017; Schwenk et al., 2017). The other sub-ability of number sense for which we identified a large but non-significant weakness in people with MD is number word knowledge. However, 3 of the 4 included studies which reported data on number word knowledge were based on a German-speaking sample. The German number-word system is not structured along the place value of numbers. That means, the order of tens and units in German number words is inverted compared to its Arabic counterparts (Klein et al., 2013). For example, number 23 is written and spoken as “three-and-twenty” instead of “twenty-three” like in English. Besides German, this so called “inversion property” is also common in other languages like Arabic or Dutch and particularly challenging for people with MD (Moeller et al., 2015; van der Ven et al., 2017). Although weaknesses in number word knowledge for people with MD have also been reported in languages without inversion property (Moura et al., 2013), we cannot completely exclude the possibility of a language bias for the large weakness found in our meta-analysis.

### Domain-General Abilities: Lack of Studies or Lack of Findings

Our lack of results regarding domain-general abilities was surprising regarding the huge body of research published in recent years about the important role of domain-general abilities for math development (Taub et al., 2008; Henik et al., 2011; Fias et al., 2013). This is because we could not consider a lot of studies since they did not meet our inclusion criteria. Since we excluded RD, we did not find substantial weaknesses in those domain-general abilities typically associated with reading difficulties and comorbid math and reading difficulties: Auditory short-term storage, retrieval fluency and phonetic coding (Peng et al., 2017; Moll et al., 2019). Also, differences in reading performance between the MD and TD group did not significantly moderate effect sizes in any of the included abilities. While this cannot be considered as a particular strength compared to people without MD, it is important to point out that people with MD perform about as well as people without MD on these domain-general abilities. As a consequence, our results contradict those of other reviews which reported medium to large weaknesses and did not exclude RD or failed to mention the exact criteria (Swanson and Jerman, 2006; Swanson et al., 2009; Johnson et al., 2010; Peng et al., 2018). Those reviews also applied liberal criteria regarding cut-offs for MD or age range, so we are not able to pinpoint the exact reason for the different results. For phonetic coding we can provide an alternative explanation for the small effect size we found in our study. This ability is associated with the development of number word knowledge and knowledge about basic arithmetic facts (de Smedt and Boets, 2010; Pollack and Ashby, 2018). Only later are facts retrieved directly from memory and people rely less on phonetic coding. Like quantity processing an age-bias is possible, as our sample of participants could have been too old to have a more severe weakness in phonetic coding. Also, Moll et al. (2019) give hints as to why we even found effects, albeit very small ones, even though RD was excluded in our study. That is, domain-specific ability computation relies stronger on language abilities than number sense and is therefore stronger associated with reading abilities. Especially, retrieving arithmetic facts from memory depends more on the processing of verbal information compared to symbolic or non-symbolic magnitude comparisons. As a result, comorbidity rates between MD and RD are four times higher when MD identification is solely based on computation than on number sense. In our meta-analysis, 97.1% of all included studies applied computation tasks to identify MD. Although we excluded severe reading difficulties, we cannot rule out the possibility that our sample of people with MD still had minor problems in reading because of the tests used to identify MD. That being said, our findings are still in line with multiple studies comparing weaknesses in MD, RD, and MD + RD on these domain-general abilities (Schuchardt et al., 2008; Willburger et al., 2008; Cirino et al., 2015; Moll et al., 2015).

#### Short-Term Working Memory

For working memory capacity our results are in accordance with multiple reviews reporting medium weaknesses in people with MD despite different inclusion criteria (e.g., Swanson and Jerman, 2006; Johnson et al., 2010; David, 2012; Peng and Fuchs, 2014; Peng et al., 2018). However, medium weaknesses in working memory capacity were also reported in people with RD and MD + RD using various stimuli and tasks (de Weerdt et al., 2013b; Willcutt et al., 2013; Cirino et al., 2015; Mähler and Schuchardt, 2016). Therefore, a general weakness in working memory capacity in people with learning difficulties seems apparent. For this reason, we do not consider the significant weaknesses in working memory capacity to be distinct enough for MD identification. We also found a medium weakness in visual-spatial short-term storage which was particularly interesting compared to the small weakness in corresponding auditory short-term storage. In recent years, several studies found weaknesses in people with MD when processing visual or visuospatial information (Landerl et al., 2009; Szûcs et al., 2013; Skagerlund and Träff, 2014). Hence, a link between number and space was assumed (Huber et al., 2015; Wong, 2017) which was transformed into a General Magnitude Deficit theory (Lourenco et al., 2016; Lourenco and Bonny, 2017; Tobia et al., 2018). According to this, numerical (e.g., dot pattern) and non-numerical magnitudes (e.g., length and time) are based upon similar neurocognitive mechanism. As a result, weaknesses in this system will lead to difficulties processing quantities correctly, which in turn make the understanding of numbers and of number relations more problematic. However, a falsification of this theory is still pending and several authors have reported contradictory results, especially for people with MD (Mussolin et al., 2011; de Visscher et al., 2018). While a General Magnitude Deficit theory seems too linear and too broad to account for complex neurodevelopmental effects and the interconnectivity between multiple brain regions (Skagerlund et al., 2016; McCaskey et al., 2017; Kucian et al., 2018), our meta-analysis gives at least further evidence that weaknesses in number and space do occur simultaneously in MD. Nevertheless, we cannot corroborate this observation with weaknesses in other visual-laden abilities like perceptual speed or visual processing since no studies based on our inclusion criteria were found.

The final ability in our study which belonged to short-term working memory is attentional control. Here, the number of included studies was small and all effect sizes were not significant. Based on the results, weaknesses for people with MD are more severe when scores are based on response time instead of accuracy which is in line with several studies (e.g., Peng et al., 2012; Wang et al., 2012). Most studies measured low-level ability inhibition, hence its effect size was similar to medium-level ability attentional control and therefore medium. We did not find enough data for other low-level abilities like shifting and updating. An explanation for the weakness in inhibition is provided by Geary et al. (2000). They suggested that people with MD have difficulties in fact retrieval because they cannot inhibit similar but nonetheless incorrect results. For example, 3 × 4 can trigger the result 8 (i.e., 2 × 4), 9 (i.e., 3 × 3), or 16 (i.e., 4 × 4), which distract from the correct result 12. However, weaknesses in attentional control and more specifically inhibition are also common in people with ADHD (Barkley, 1997). ADHD was not excluded in our sample since most studies did not control for it. Thus only 3 out of 7 studies reporting data on inhibition excluded ADHD. The findings of our meta-analysis do not support Peng et al. (2018), who reported only a small weakness for inhibition and found instead a large one for updating and shifting in people with MD. Two explanations for this difference in findings between both reviews emerge. First, it is unclear if effect sizes in Peng et al. (2018) are based on accuracy, response time, or both. Since this meta-analysis found smaller effect sizes in accuracy and larger ones in response time the type of score seems to affect the severity of deficits. Second, deficits in inhibition, updating and shifting have also been found for people with isolated RD and comorbid MD+RD (van der Sluis et al., 2004; Booth et al., 2010). Especially shifting and updating seem to be more related to RD than inhibition (Peng et al., 2013; Moura et al., 2014). Since Peng et al. (2018) did not report their criteria used to exclude RD it is unclear if their findings were corroborated by reading difficulties.

#### Other Abilities

The last two high-level abilities for which studies reported data are reaction and decision speed and fluid reasoning. We did not discuss fluid reasoning much in the introduction and not mentioned reaction and decision speed at all because reported data on both abilities are more a by-product than the main focus of its respective studies. Reaction and decision speed means to react and decide quickly to the onset of a simple stimulus (e.g., press a button when hearing a sound). In studies about MD it is mostly measured as a part of an extensive test battery. For fluid reasoning, most studies controlled for low IQ which in turn affects the overall effect. Also, it is unclear if the respective IQ test was also used to control for low IQ which would result in higher average IQs and smaller variances in the MD and TD group. For both abilities the number of studies reporting data is small. We found a small and non-significant weakness for reaction and decision speed which supports findings by Szûcs et al. (2013) and Raddatz et al. (2017) in that very simple non-numerical tasks do not differ well between people with and without MD. The average effect size for fluid reasoning was medium in our meta-analysis. Considering the methodological uncertainties with IQ scores and the fact that other reviews reported mixed findings for fluid reasoning, we refrain from comparing our results with theirs and from deriving any conclusion about the role of IQ for MD.

### Limitations

First, the number of studies for each outcome varied considerably and dropped with increasing specificity of outcomes (e.g., from high-level to low-level ability). While meta-analyses can be performed with no more than 2 studies, results are usually more robust and less prone to publication bias when more studies are included (Lin, 2018). Especially when random effects and a certain degree of heterogeneity between and within studies are expected, effect sizes based on the data of few studies need to be interpreted cautiously (Valentine et al., 2010). To solve this problem, we applied meta-regressions to examine the influence of moderating variables. However, also those measures are accompanied with various issues (e.g., low power for small sample size, no random assignment of studies to moderators) and therefore only give hints on possible data problems (Oxman and Guyatt, 1992; Walker et al., 2008; Wood and Eagly, 2009). Most importantly, we have therefore used strict inclusion criteria in accordance with DSM and ICD to derive valid results. On the one hand, those criteria were responsible for our small sample of studies. On the other hand, they led to a representative sample of MD without other corrupting conditions (comorbidities) or issues (broad age range and high cut-off value) usually accompanied with more liberal criteria. Nevertheless, Egger’s test also indicated a publication bias in data set AC + TC and in data set RT + TC for high-level ability mathematics and medium-level ability number sense, respectively. Although this does not fundamentally contradict our findings, a small-study effect for those abilities is possible.

Second, across all data sets, heterogeneity *I*^2^ (Higgins and Thompson, 2002) was substantial and on average 57.5% (AC − TC: 53.6%, AC + TC: 52.6%, RT + TC: 66.4%). Although descriptive information of all included studies indicated a homogenous data set, there is inconsistency in the data which was neither accounted for by our statistical model nor further explained by any moderator we applied. Also, heterogeneity was larger for response time scores than for accuracy scores. From a methodological perspective, small samples tend to increase heterogeneity (IntHout et al., 2015). Also type of scoring could have affected heterogeneity. Data set RT + TC had the highest heterogeneity, the smallest sample size and studies varied considerably in how they measured response time scores (e.g., total time and time for correct answers), detected outliers, or transformed data (e.g., log transformation). Those variations may have affected effect sizes, hence led to greater inconsistencies between studies in data set RT + TC. From a theoretical perspective, Kaufmann et al. (2013) defined heterogeneity as a feature of MD. According to them, people with MD have a core weakness in domain-specific abilities which can be accompanied by weaknesses in domain-general abilities as well. However, the type and severity of all those weaknesses varies depending on the neurofunctional and behavioral development of each individual person with MD. As a result, differences between people with and without MD are less moderated by study design. Instead, heterogeneous manifestations of MD exist within every MD sample due to interindividual neurodevelopmental differences (Zhang et al., 2017; McCaskey et al., 2018). This perspective is also in accordance with a vast research body about different subtypes of MD (Landerl et al., 2004, 2009; Gold et al., 2013; Bartelet et al., 2014; Cirino et al., 2015; Shin and Bryant, 2015; Träff et al., 2017). However, such research questions can only be answered by using controlled studies. While we do not deny the possibility of different subtypes of MD to explain the overall heterogeneity, we can neither test nor control for it. The scope of this meta-analysis was to summarize strengths and weaknesses of MD based on clinical criteria.

Third, ADHD was not deliberately excluded by us. About 10–20% of people with MD also have ADHD or show symptoms thereof (Gross-Tsur et al., 1996; Fortes et al., 2016). Kuhn et al. (2016) have shown that the profile of people with MD + ADHD is a combination of the distinct weaknesses of people with MD or ADHD. Only 16 out of 34 studies of our sample controlled for ADHD. And those who did relied mostly on existing information instead of applying any additional measures. As a result, studies including comorbid cases of MD + ADHD are possible and effect sizes for ADHD-specific outcomes like attentional control were interpreted carefully.

Fourth, studies that did not control for reading difficulties were excluded rather than being coded and used as moderator. We refrained from using a subgroup analysis to compare effect sizes of studies that excluded RD to those that did not for two reasons. First, for studies that did not exclude RD we could not make any assumptions about the distribution of reading performance within each sample. Especially if reading performance was not measured people with RD could have been in the sample thus co-founding the subgroup analysis. Second, subgroup analyses have been criticized for their low statistical power because categories with a different amount of studies and varying study quality are being compared (Oxman and Guyatt, 1992; Walker et al., 2008). While meta-analyses are based on a systematic literature following clear inclusion criteria, subgroup analyses are only observational (i.e., cross-sectional studies) because neither are the studies randomly assigned to each group nor are any moderating variables being controlled for (Higgins and Green, 2011). While this problem also applies to meta-regressions, it is especially pronounced for subgroup analyses. Since this meta-analysis had already to deal with low sample sizes because of our conservative inclusion criteria, we decided that the statistical power of any subgroup analysis would have been too low to allow any conclusions. This also applies to ADHD for which we did not perform a subgroup analysis either.

Fifth, we did not find any particular strength for the MD group based on the effect size. There were many small effect sizes which we refrained from classifying as a particular “strength” or “non-weakness.” By using the terms “strength” and “weakness” we tried to emphasize our exploratory coding approach. Since we did not restrict our coding scheme to certain abilities, we could not rule out that a medium to large effects sizes in favor of the MD group in any ability was theoretically possible.

### Implications and Conclusion

We identified a cognitive profile of domain-specific and domain-general deficits of MD which is based on criteria according to the DSM and ICD. Whereas DSM and ICD only describe in a very general way which abilities are affected in MD, we found a distinct set of well operationalized abilities which evidently differ best between groups of children (8–12 years old) with and without MD. These were: Calculation (AC − TC), fact retrieval (AC + TC), quantity processing (AC + TC), quantity-number linking (RT + TC), numerical relations (AC − TC), and visual-spatial short-term storage (AC − TC). This profile helps experts working with DSM or ICD to revise their general diagnostic procedures und treatment plans. Also, for clinical guidelines about diagnostic and treatment of MD, our profile serves as high quality evidence-based information derived by a systematic literature and meta-analysis. However, we also want to point out that this profile is based on average group differences and therefore cannot account for the heterogenous development of MD in individuals.

Our meta-analysis was the first in many aspects. We used stringent inclusion criteria according to DSM and ICD, summarized domain-specific abilities in a systematic way, applied a multi-level coding scheme and differentiated scoring types. While this approach was very laborious and needed many adjustments, it resulted in a comprehensive profile about MD which has various implications for further research. Most importantly, when controlling for reading difficulties MD is not accompanied by substantial weaknesses in those domain-general abilities which are typically assigned to reading (i.e., phonetic coding, naming facility, and auditory short-term storage). Also, visual-spatial short-term storage is more affected in MD compared to auditory short-term storage which emphasizes the notion that number and space are interlinked and overall important for math development. In the past, multiple fMRI studies have reported neuronal activity in areas allocated to domain-general abilities when doing arithmetic and even suggested a neuronal network of domain-specific and domain-general abilities (Kucian, 2016; Peters and de Smedt, 2018). If and how this applies to MD is still unknown. Unfortunately, also our meta-analysis could not derive a distinct pattern of strengths and weaknesses in domain-general abilities since most studies about domain-general abilities did not meet our stringent inclusion criteria. A pressing need for high quality studies investigating those abilities is obvious.

## Data Availability Statement

The datasets presented in this study can be found in online repositories. The names of the repository/repositories and accession number(s) can be found at: https://osf.io/sbfcz/.

## Author Contributions

SH and GS-K contributed to conception and design of the study. SH performed the literature search, coding, statistical analysis, and wrote the first draft of the manuscript. GS-K revised first draft of the manuscript. Both authors contributed to the article and approved the submitted version.

## Conflict of Interest

The authors declare that the research was conducted in the absence of any commercial or financial relationships that could be construed as a potential conflict of interest.

## Publisher’s Note

All claims expressed in this article are solely those of the authors and do not necessarily represent those of their affiliated organizations, or those of the publisher, the editors and the reviewers. Any product that may be evaluated in this article, or claim that may be made by its manufacturer, is not guaranteed or endorsed by the publisher.
