# Supplementary material for: The Cognitive Profile of Math Difficulties: A Meta-Analysis Based on Clinical Criteria
Source: Front Psychol. 2022 Mar 11;13:842391. doi: 10.3389/fpsyg.2022.842391 (PMC8962618; doi:10.3389/fpsyg.2022.842391)
Supplement: Supplementary file 1 [file Data_Sheet_1.docx]

Supplementary Material

# Supplementary Text

Search term in English:

(dyscalculi* OR "math* disorder*" OR "math* disabilit*" OR "math* difficult*" OR "math* learning disorder*" OR "math* learning disabilit*" OR "math* learning difficult*" OR "arithmetic* disorder*” OR "arithmetic* disabilit*" OR "arithmetic* difficult*" OR "arithmetic* learning disorder*" OR "arithmetic* learning disabilit*" OR "arithmetic* learning difficult*" OR "impairment* in math*" OR "developmental acalculia" OR "disorder* of arithmetic* skill*" OR "low math* achievement*" OR "low arithmetic* achievement*" OR "disorder* in math*" OR "difficul* in math*" OR "disabilit* in math*" OR "disorder* in arithmetic*" OR "difficul* in arithmetic*" OR "disabilit* in arithmetic*" OR "math* LD") AND (differ OR differs OR differed OR difference* OR disting* OR discrim* OR contrast* OR gap* OR compar* OR "experimental group*" OR "control group*" OR "typical* achiev*" OR "normal* achiev*" OR "typical* develop*" OR "normal develop*").

Search term in German:

(Rechenstörung* OR Dyskalkul* OR Rechenschwäche* OR Zahlenblindheit OR Arithmasthenie OR "Schwierigkeit* im Rechnen" OR "Problem* im Rechnen" OR "Schwierigkeit* beim Rechnen" OR "Problem* beim Rechnen" OR Rechenschwierigkeit* OR Rechenproblem* OR "des Rechnens") AND (Unterschied* OR unterscheid* OR Differenz* OR Diskrepan* OR trennt OR vergleich* OR Versuchsgruppe* OR Kontrollgruppe* OR Experimentalgruppe*)

# Supplementary Tables

**Table S1**

*Coding Scheme With Descriptions of Outcomes and Task Examples*

| Outcome (HA/MA/LA)^a^ | Studies (included) | | | |  | Description | Example |
| --- | --- | --- | --- | --- | --- | --- | --- |
|  | AC+  TC | AC-  TC | RT+  TC | RT-  TC |  |  |  |
| Domain-specific abilities | | | | | | | |
| Mathematics | 9  (9) | 16  (15) | 2  (0) | 14  (13) |  | Solve mathematical or mathematical-related problems using computation, reasoning and processing of numbers and magnitudes. |  |
| Computation | 5  (5) | 8  (7) | 1  (0) | 3  (3) |  | Solve arithmetical problems. |  |
| Calculation | 5  (5) | 0 | 1  (0) | 0 |  | Solve arithmetical problems by completing basic arithmetic operations. | 818 + 104 |
| Fact retrieval | 0 | 7  (6) | 0 | 2  (0) |  | Solve arithmetical problems by retrieval of facts from memory. | 3 x 5 |
| Estimation | 0 | 1  (0) | 0 | 1  (0) |  | Solve arithmetical problems by estimating the results. Usually arithmetical problems are too complex to be solved by calculation within the given time limit or by fact retrieval, respectively. | 497 / 27.  Which number is closer to the result: 20 or 40? |
| Math reasoning | 3  (3) | 0 | 0 | 0 |  | Applicate mathematical knowledge to solve complex, unfamiliar, or multistep problems. | Word problems (e.g. Peter has 5 apples He gives Jane 1 apple and Paul 2 apples. How many apples does Peter have left?)  Decide if two arithmetical problems can be solved by the same approach. |
| Number sense | 6  (6) | 13  (13) | 1  (0) | 13  (12) |  | Understand quantities and numbers. |  |
| Number word knowledge | 6  (6) | 0 | 1  (0) | 0 |  | Knowledge of number words (2 = “two”) | Transcoding (e.g. Number 17 is read out (i.e. “seventeen”). Write it down in Arabic digits (i.e. 17). |
| Quantity processing | 0 | 6  (6) | 0 | 4  (4) |  | Differentiate between quantities of objects without counting. | Non-symbolic comparison (e.g. A set of 4 dots is presented next to a set of 7 dots. Which magnitude is larger?) |
| Quantity-number linking | 0 | 7  (7) | 0 | 8  (7) |  | Understand the relationship between quantities, numbers and number words (●●● = 3 = “three”). | Subitizing (e.g. 2 black dots are presented on a white screen for 500 ms. Say the amount of dots immediately after presentation.)  Dot enumeration (e.g. 5 black dots are presented on a white screen for 500 ms. Say the amount of dots immediately after presentation.) |
| Numerical relations | 6  (6) | 4  (4) | 0 | 5  (5) |  | Know the correct sequence of numbers (i.e. 1, 2, 3) and apply it to compare the values of numbers. | Symbolic comparison (e.g. Number 4 is presented next to number 7. Which number is larger?)  Number line (e.g. A number line from 0 to 100 is presented. Locate number 40 on the number line.)  Counting (e.g. Count aloud from 10 to 20.) |
| Part-part-whole | 0 | 1  (0) | 0 | 0 |  | Divide magnitudes (objects or numbers) into smaller subsets or vice versa. | Number sets (e.g. A set of 2 dots and number 3 are presented next to number 7. Which magnitude is larger?) |
| Reading and writing | 3  (3) | 6  (6) | 0 | 3  (3) |  |  |  |
| Phonetic coding | 3  (3) | 0 | 0 | 0 |  | Manipulate phonemes, syllables, or onset-rimes. | Delete a specific sound from a word (e.g. say “cat” without the /k/). |
| Retrieval fluency | 0 | 5  (5) | 0 | 3  (3) |  | Retrieve verbal or nonverbal information from long-term memory quickly. |  |
| Naming facility | 0 | 5  (5) | 0 | 3  (3) |  | Retrieve the name of well-known visual stimuli (letters, objects, colors) from long-term memory quickly. | A list of letters is presented or letters are presented one after another. Name each letter as quickly as possible. |
| Word fluency | 0 | 1  (0) | 0 | 0 |  | Produce words with a similar semantic or phonological feature. | Say as many words as possible which begin with the letter “k”. |
| Reading decoding | 0 | 2  (0) | 0 | 0 |  | Identify words correctly. | Read a list of words aloud. |
| Domain-general abilities | | | | | | | |
| Short-term working memory | 16  (16) | 5  (5) | 1  (0) | 5  (5) |  |  |  |
| Auditory short-term storage | 9  (9) | 0 | 1  (0) | 0 |  | Store auditory information temporarily. | Auditory forward span (e.g. A letter sequence is presented (e.g. F-T-C-A). Recall the letters in the given order (i.e. F-T-C-A).)  Auditory recall (e.g. A letter sequence is presented (e.g. F-T-C-A). Recall the letters in any order (e.g. T-A-F-C).) |
| Visual-spatial short-term storage | 9  (9) | 0 | 1  (0) | 0 |  | Store visuospatial information temporarily. | Visuospatial forward span (Corsi block-tapping test: A set of blocks is presented. 4 blocks are tapped one after another. Recall the blocks and their position and tap the blocks in the same order.  Visuospatial forward recall (e.g. A set of blocks is presented. 4 blocks are tapped one after another. Recall the blocks and tap the blocks in any order.) |
| Working memory capacity | 13  (13) | 0 | 1  (0) | 0 |  | Store and process information (same or different) simultaneously. | Backward span (e.g. A letter sequence is presented (e.g. F-T-C-A). Recall the letters in the reverse order (i.e. A-C-T-F).)  Complex span (e.g. A set of sentences is read out (e.g. The sky is green. A ball is round. Ice is cold). After each sentence, decide if the sentence is true or false. After the complete set, recall the last word of each sentence in the given order (i.e. green-round-cold).)  Dual task (e.g. A set of numbers and letters, each in random order (e.g. 3-1-4-2 and C-B-A-D, respectively), is presented. Recall each set in sequential order (i.e. 1-2-3-4 and A-B-C-D, respectively).) |
| Attentional control | 0 | 5  (5) | 0 | 5  (5) |  | Monitor, adapt and regulate cognitive performance in reaction to changing task settings |  |
| Inhibition | 0 | 4  (4) | 0 | 5  (5) |  | Inhibit a prepotent response deliberately. | Go/no-go (e.g. Press a key when a specific stimulus appears (go-condition).)  Random generation (e.g. Write numbers 1 to 10 in random order (e.g. 4-1-9-7).)  Stop signal task (e.g. An arrow pointing to the left or right is presented. If there’s no sound after presentation press left or right depending on the direction of the arrow. If there’s a sound don’t press any button.)  Stroop task (e.g. The word “blue” is presented in color yellow. Name the color of the word (i.e. yellow) and not the content of the word (i.e. red).)  Flanker task (e.g. 4 arrows point to the right. A 5th arrow in the middle of those 4 arrows points to the left. Press left or right depending on the direction of the middle arrow.) |
| Shifting | 0 | 1  (0) | 0 | 1  (0) |  | Shift between tasks. | Trail making B (e.g. Connect numbers and letters alternately in sequential order (e.g. 1-A-2-B-3-C etc.).)  A number (e.g. 2) within an object (e.g. triangle) is presented. If the color of number and object is red name the number. If the color of number and object is blue name the object. |
| Updating | 0 | 1  (0) | 0 | 0 |  | Update task-relevant information in memory. | A set of numbers with varying length is read out. Name the last 3 numbers.  Numbers are presented one after another. Press a button if the presented number is the same number presented 2 numbers previously. |
| Processing speed | 0 | 2  (0) | 0 | 1  (0) |  | Encode information quickly and perform simple cognitive tasks based on that information fast. |  |
| Perceptual speed | 0 | 2  (0) | 0 | 1  (0) |  | Compare visual stimuli for differences and similarities. | Trail making A (e.g. Letters from A to Z are randomly distributed over a sheet of paper. Connect the letters in sequential order by drawing lines (i.e. A-B-C-D etc.).)  Coding (e.g. Numbers from 1 to 9 are paired with a unique symbol each. Several numbers from 1 to 9 without its corresponding symbols are presented. Copy the corresponding symbols to the numbers as fast as possible.)  Visual comparison (e.g. Locate and circle a target symbol within a row of 6 different symbols.) |
| Reaction and decision speed | 0 | 2  (0) | 0 | 3  (3) |  | React quickly when a simple condition is met or quickly decide which condition is met and react accordingly |  |
| Simple reaction time | 0 | 0 | 0 | 1  (0) |  | React quickly to the onset of a stimulus | A blank screen is presented. Press a button as fast as possible when a dot appears. |
| Choice reaction time | 0 | 2  (0) | 0 | 2  (0) |  | Make a simple choice quickly and react accordingly. | A blank screen is presented. Press the left or right button as fast as possible when a dot appears left or right, respectively. |
| Visual processing | 1  (0) | 0 | 0 | 0 |  | Perceive, discriminate, manipulate, and recall non-linguistic images to solve problems. |  |
| Spatial scanning | 1  (0) | 0 | 0 | 0 |  | Accurately survey a spatial field. | Solve a maze. |
| Learning efficiency | 1  (0) | 0 | 0 | 0 |  | Learn and store new information. |  |
| Associative memory | 1  (0) | 0 | 0 | 0 |  | Learn and remember the relationship between unrelated stimuli. | First, words are simultaneously presented with pictures. After that, recall the correct word by seeing its corresponding picture. |
| Comprehension-knowledge | 2  (0) | 0 | 0 | 0 |  | Acquire and apply culture-dependent knowledge. |  |
| Language development | 1  (0) | 0 | 0 | 0 |  | Understand spoken language. | Sentences where each is missing a word are read out loud. Complete each sentence correctly. |
| Lexical knowledge | 1  (0) | 0 | 0 | 0 |  | Understand the meaning of words without context (i.e. vocabulary). | Words are spoken out loud. Match each word with the correct picture that describes it. |
| Fluid reasoning | 3  (3) | 0 | 0 | 0 |  | Solve unfamiliar problems where previously learned solutions for familiar problems do not work. |  |
| Induction | 2  (0) | 0 | 0 | 0 |  | Discover underlying patterns to solve a problem (i.e. inductive reasoning). | Matrix reasoning (e.g. Find a pattern in a series of figures.)  Find the relationship between groups of word (e.g. football, tennis, basketball = sports) |
| General sequential reasoning | 1  (0) | 0 | 0 | 0 |  | Apply known principles to solve a problem (i.e. deductive reasoning). | Syllogistic-reasoning tasks (e.g. All cats have four legs. I have a cat. My cat has four legs.) |

*Note.* HA = high-level ability; MA = medium-level ability; LA = low-level ability; Studies = number of studies after literature search; included = number of studies included in the meta-analysis; AC = accuracy; RT = response time; TC = time constraints.

^a^Subordination of outcome reflects level, i.e. high-level ability (no indent), medium-level ability (medium indent), and low-level ability (large indent).

**Table S2**

*Extended Results of Random-Effects Model with RVE for Data Sets AC-TC, AC+TC and RT+TC*

| Outcome (HA/MA/LA)^a^ |  |  |  |  |  |  |  |  | Heterogeneity | |
| --- | --- | --- | --- | --- | --- | --- | --- | --- | --- | --- |
|  | ST | ES | *df* | *g* | *SE* | *CI95* | *t* | *p* | *I²* | *τ²* |
| Mathematics |  |  |  |  |  |  |  |  |  |  |
| AC-TC | 9 | 45 | 7.05 | 1.04 | 0.17 | [0.63, 1.45] | 5.99 | .001 | 52.68 | 0.09 |
| AC+TC | 16 | 72 | 13.26 | 0.78 | 0.12 | [0.52, 1.04] | 6.46 | .000 | 47.76 | 0.09 |
| RT+TC | 13 | 56 | 10.47 | 0.71 | 0.14 | [0.39, 1.03] | 4.93 | .001 | 66.78 | 0.22 |
| Computation |  |  |  |  |  |  |  |  |  |  |
| AC-TC | 5 | 13 | 2.49 | 1.19 | 0.32 | [0.04, 2.35] | 3.72 | .046 | 49.77 | 0.08 |
| AC+TC | 8 | 26 | 5.55 | 0.81 | 0.19 | [0.35, 1.28] | 4.37 | .006 | 50.47 | 0.10 |
| RT+TC | 3 | 6 | 1.71 | 0.38 | 0.43 | [-1.79, 2.54] | 0.88 | .483 | 67.49 | 0.23 |
| Calculation |  |  |  |  |  |  |  |  |  |  |
| AC-TC | 5 | 13 | 2.78 | 1.15 | 0.24 | [0.33, 1.96] | 4.70 | .022 | 63.75 | 0.15 |
| Fact retrieval |  |  |  |  |  |  |  |  |  |  |
| AC+TC | 7 | 22 | 4.53 | 0.87 | 0.22 | [0.27, 1.46] | 3.86 | .014 | 56.35 | 0.13 |
| Math reasoning |  |  |  |  |  |  |  |  |  |  |
| AC-TC | 3 | 3 | 1.78 | 1.29 | 0.11 | [0.73, 1.84] | 11.20 | .012 | 49.77 | 0.08 |
| Number sense |  |  |  |  |  |  |  |  |  |  |
| AC-TC | 6 | 29 | 4.57 | 0.87 | 0.17 | [0.41, 1.33] | 5.00 | .005 | 49.77 | 0.08 |
| AC+TC | 13 | 46 | 9.30 | 0.75 | 0.16 | [0.39, 1.11] | 4.75 | .001 | 50.47 | 0.10 |
| RT+TC | 12 | 50 | 8.95 | 0.77 | 0.15 | [0.44, 1.10] | 5.28 | .001 | 67.49 | 0.23 |
| Number word knowledge |  |  |  |  |  |  |  |  |  |  |
| AC-TC | 4 | 8 | 2.52 | 0.84 | 0.20 | [0.13, 1.55] | 4.23 | .034 | 63.75 | 0.15 |
| Quantity processing |  |  |  |  |  |  |  |  |  |  |
| AC+TC | 6 | 25 | 3.87 | 0.69 | 0.10 | [0.42, 0.96] | 7.18 | .002 | 56.35 | 0.13 |
| RT+TC | 4 | 22 | 2.23 | 0.27 | 0.08 | [-0.04, 0.57] | 3.45 | .064 | 65.26 | 0.20 |
| Quantity-number linking |  |  |  |  |  |  |  |  |  |  |
| AC+TC | 7 | 9 | 3.56 | 0.69 | 0.33 | [-0.28, 1.65] | 2.08 | .114 | 56.35 | 0.13 |
| RT+TC | 7 | 12 | 4.17 | 0.77 | 0.10 | [0.51, 1.03] | 8.00 | .001 | 65.26 | 0.20 |
| Numerical relations |  |  |  |  |  |  |  |  |  |  |
| AC-TC | 6 | 19 | 4.58 | 0.90 | 0.17 | [0.47, 1.34] | 5.45 | .004 | 63.75 | 0.15 |
| AC+TC | 5 | 12 | 3.19 | 0.87 | 0.28 | [0, 1.74] | 3.08 | .050 | 56.35 | 0.13 |
| RT+TC | 5 | 16 | 3.66 | 1.03 | 0.27 | [0.24, 1.83] | 3.76 | .023 | 65.26 | 0.20 |
| Reading and writing |  |  |  |  |  |  |  |  |  |  |
| AC-TC | 3 | 3 | 1.88 | 0.33 | 0.04 | [0.13, 0.53] | 7.63 | .020 | 52.68 | 0.09 |
| AC+TC | 6 | 20 | 3.68 | 0.14 | 0.12 | [-0.2, 0.49] | 1.21 | .297 | 47.76 | 0.09 |
| RT+TC | 3 | 8 | 1.94 | 0.00 | 0.15 | [-0.67, 0.67] | 0.00 | .999 | 66.78 | 0.22 |
| Phonetic coding |  |  |  |  |  |  |  |  |  |  |
| AC-TC | 3 | 3 | 1.91 | 0.33 | 0.04 | [0.14, 0.52] | 7.94 | .018 | 49.77 | 0.08 |
| Retrieval fluency |  |  |  |  |  |  |  |  |  |  |
| AC+TC | 5 | 15 | 2.65 | 0.04 | 0.05 | [-0.14, 0.23] | 0.80 | .491 | 50.47 | 0.10 |
| RT+TC | 3 | 8 | 1.94 | 0.00 | 0.15 | [-0.67, 0.67] | 0.00 | .998 | 67.49 | 0.23 |
| Naming facility |  |  |  |  |  |  |  |  |  |  |
| AC+TC | 5 | 13 | 2.63 | 0.03 | 0.07 | [-0.21, 0.28] | 0.46 | .683 | 56.35 | 0.13 |
| RT+TC | 3 | 8 | 1.94 | 0.05 | 0.18 | [-0.75, 0.84] | 0.25 | .825 | 65.26 | 0.20 |
| Short-term working memory |  |  |  |  |  |  |  |  |  |  |
| AC-TC | 16 | 83 | 12.52 | 0.56 | 0.07 | [0.41, 0.71] | 8.18 | .000 | 52.68 | 0.09 |
| AC+TC | 5 | 11 | 2.83 | 0.23 | 0.08 | [-0.02, 0.48] | 3.05 | .060 | 47.76 | 0.09 |
| RT+TC | 5 | 16 | 3.48 | 0.58 | 0.15 | [0.13, 1.02] | 3.84 | .024 | 66.78 | 0.22 |
| Auditory short-term storage |  |  |  |  |  |  |  |  |  |  |
| AC-TC | 9 | 30 | 7.16 | 0.36 | 0.07 | [0.21, 0.51] | 5.50 | .001 | 49.77 | 0.08 |
| Visual-spatial short-term storage |  |  |  |  |  |  |  |  |  |  |
| AC-TC | 9 | 24 | 4.80 | 0.66 | 0.12 | [0.34, 0.98] | 5.36 | .003 | 49.77 | 0.08 |
| Working memory capacity |  |  |  |  |  |  |  |  |  |  |
| AC-TC | 13 | 27 | 6.89 | 0.61 | 0.11 | [0.34, 0.88] | 5.41 | .001 | 49.77 | 0.08 |
| Attentional control |  |  |  |  |  |  |  |  |  |  |
| AC+TC | 5 | 11 | 2.84 | 0.23 | 0.08 | [-0.02, 0.48] | 3.06 | .059 | 50.47 | 0.10 |
| RT+TC | 5 | 16 | 3.48 | 0.58 | 0.15 | [0.13, 1.02] | 3.84 | .024 | 67.49 | 0.23 |
| Inhibition |  |  |  |  |  |  |  |  |  |  |
| AC+TC | 4 | 7 | 2.03 | 0.16 | 0.05 | [-0.06, 0.38] | 3.06 | .091 | 56.35 | 0.13 |
| RT+TC | 5 | 13 | 3.23 | 0.56 | 0.16 | [0.06, 1.06] | 3.39 | .038 | 65.26 | 0.20 |
| Reaction and decision speed |  |  |  |  |  |  |  |  |  |  |
| RT+TC | 3 | 3 | 1.25 | 0.38 | 0.18 | [-1.05, 1.81] | 2.14 | .236 | 66.78 | 0.22 |
| Fluid reasoning |  |  |  |  |  |  |  |  |  |  |
| AC-TC | 3 | 5 | 1.31 | 0.58 | 0.12 | [-0.3, 1.45] | 4.86 | .086 | 52.68 | 0.09 |

*Note.* Positive effect sizes indicate a higher accuracy or faster response time in the TD group. AC = accuracy; RT = response time; TC = time constraints; HA = high-level ability; MA = medium-level ability; LA = low-level ability; ST = number of studies; ES = number of effect sizes; *g* = Hedges’ *g*; *SE* = standard error; *CI95* = 95% confidence interval.

^a^Subordination of outcome reflects content, i.e., high-level ability (no indent), medium-level ability (medium indent), and low-level ability (large indent). Only those outcomes are shown for which the analysis could be performed.

Table S3

*Risk of Bias Analyses Across Studies*

| Outcome (HA/MA/LA)^a^ | ST | ES | *df* | *b* | *SE* | *CI95* | *t* | *p* |
| --- | --- | --- | --- | --- | --- | --- | --- | --- |
| Egger | | | | | | | | |
| Mathematics |  |  |  |  |  |  |  |  |
| AC-TC | 9 | 45 | 2.15 | -0.50 | 2.43 | [-10.28, 9.28] | -0.20 | .856 |
| AC+TC | 16 | 72 | 4.45 | 3.12 | 1.05 | [0.31, 5.93] | 2.96 | .036 |
| RT+TC | 13 | 56 | 6.04 | 3.21 | 3.58 | [-5.53, 11.94] | 0.90 | .404 |
| Computation |  |  |  |  |  |  |  |  |
| AC-TC | 5 | 13 | 2.41 | -2.27 | 3.65 | [-15.67, 11.14] | -0.62 | .588 |
| AC+TC | 8 | 26 | 2.49 | 3.19 | 1.33 | [-1.58, 7.96] | 2.40 | .114 |
| RT+TC | 3 | 6 | 1.00 | -3.25 | 8.24 | [-107.97, 101.48] | -0.39 | .761 |
| Calculation |  |  |  |  |  |  |  |  |
| AC-TC | 5 | 13 | 2.25 | -0.88 | 2.84 | [-11.89, 10.13] | -0.31 | .783 |
| Fact retrieval |  |  |  |  |  |  |  |  |
| AC+TC | 7 | 22 | 2.38 | 3.34 | 1.22 | [-1.19, 7.87] | 2.74 | .093 |
| Math reasoning |  |  |  |  |  |  |  |  |
| AC-TC | 3 | 3 | 1.00 | 0.61 | 1.06 | [-12.84, 14.06] | 0.58 | .667 |
| Number sense |  |  |  |  |  |  |  |  |
| AC-TC | 6 | 29 | 1.36 | 3.76 | 3.75 | [-22.28, 29.81] | 1.00 | .460 |
| AC+TC | 13 | 46 | 6.14 | 3.81 | 1.98 | [-1.01, 8.63] | 1.92 | .102 |
| RT+TC | 12 | 50 | 6.03 | 5.53 | 2.10 | [0.41, 10.65] | 2.64 | .038 |
| Number word knowledge |  |  |  |  |  |  |  |  |
| AC-TC | 4 | 8 | 1.43 | 10.21 | 5.55 | [-25.68, 46.10] | 1.84 | .255 |
| Quantity processing |  |  |  |  |  |  |  |  |
| AC+TC | 6 | 25 | 1.77 | 4.39 | 1.74 | [-4.11, 12.88] | 2.53 | .143 |
| RT+TC | 4 | 22 | 1.82 | -2.19 | 0.70 | [-5.52, 1.14] | -3.11 | .101 |
| Quantity-number linking |  |  |  |  |  |  |  |  |
| AC+TC | 7 | 9 | 2.01 | 2.20 | 3.74 | [-13.84, 18.24] | 0.59 | .616 |
| RT+TC | 7 | 12 | 1.90 | 4.38 | 2.69 | [-7.82, 16.58] | 1.63 | .252 |
| Numerical relations |  |  |  |  |  |  |  |  |
| AC-TC | 6 | 19 | 1.28 | 3.07 | 3.58 | [-24.43, 30.56] | 0.86 | .522 |
| AC+TC | 5 | 12 | 2.16 | 4.89 | 3.04 | [-7.29, 17.06] | 1.61 | .240 |
| RT+TC | 5 | 16 | 2.11 | 8.05 | 1.66 | [1.25, 14.86] | 4.84 | .036 |
| Reading and writing |  |  |  |  |  |  |  |  |
| AC-TC | 3 | 3 | 1.00 | 0.25 | 1.13 | [-14.15, 14.65] | 0.22 | .861 |
| AC+TC | 6 | 20 | 1.10 | 2.30 | 0.87 | [-6.75, 11.35] | 2.63 | .213 |
| RT+TC | 3 | 8 | 1.02 | 4.34 | 6.41 | [-73.90, 82.57] | 0.68 | .620 |
| Phonetic coding |  |  |  |  |  |  |  |  |
| AC-TC | 3 | 3 | 1.00 | 0.22 | 1.08 | [-13.54, 13.97] | 0.20 | .874 |
| Retrieval fluency |  |  |  |  |  |  |  |  |
| AC+TC | 5 | 15 | 1.22 | 1.00 | 0.58 | [-3.88, 5.88] | 1.73 | .299 |
| RT+TC | 3 | 8 | 1.02 | 4.33 | 6.40 | [-73.79, 82.46] | 0.68 | .620 |
| Naming facility |  |  |  |  |  |  |  |  |
| AC+TC | 5 | 13 | 1.20 | 0.99 | 0.84 | [-6.26, 8.24] | 1.18 | .422 |
| RT+TC | 3 | 8 | 1.02 | 5.22 | 6.48 | [-73.86, 84.30] | 0.81 | .566 |
| Short-term working memory |  |  |  |  |  |  |  |  |
| AC-TC | 16 | 83 | 3.19 | 1.41 | 1.90 | [-4.43, 7.26] | 0.75 | .507 |
| AC+TC | 5 | 11 | 2.30 | 2.41 | 4.01 | [-12.83, 17.66] | 0.60 | .601 |
| RT+TC | 5 | 16 | 2.03 | -0.10 | 2.61 | [-11.15, 10.94] | -0.04 | .972 |
| Auditory short-term storage |  |  |  |  |  |  |  |  |
| AC-TC | 9 | 30 | 1.67 | 1.87 | 1.05 | [-3.60, 7.34] | 1.78 | .240 |
| Visual-spatial short-term storage |  |  |  |  |  |  |  |  |
| AC-TC | 9 | 24 | 2.97 | 9.52 | 7.60 | [-14.79, 33.83] | 1.25 | .300 |
| Working memory capacity |  |  |  |  |  |  |  |  |
| AC-TC | 13 | 27 | 2.59 | -1.57 | 1.61 | [-7.19, 4.06] | -0.97 | .413 |
| Attentional control |  |  |  |  |  |  |  |  |
| AC+TC | 5 | 11 | 2.30 | 2.40 | 4.02 | [-12.90, 17.69] | 0.60 | .604 |
| RT+TC | 5 | 16 | 2.04 | -0.10 | 2.60 | [-11.11, 10.91] | -0.04 | .972 |
| Inhibition |  |  |  |  |  |  |  |  |
| AC+TC | 4 | 7 | 1.90 | 0.86 | 2.25 | [-9.32, 11.05] | 0.38 | .739 |
| RT+TC | 5 | 13 | 2.24 | -0.90 | 2.93 | [-12.32, 10.52] | -0.31 | .786 |
| Reaction and decision speed |  |  |  |  |  |  |  |  |
| RT+TC | 3 | 3 | 1.00 | 8.33 | 17.60 | [-215.33, 232.00] | 0.47 | .719 |
| Fluid reasoning |  |  |  |  |  |  |  |  |
| AC-TC | 3 | 5 | 1.00 | -1.42 | 0.30 | [-5.17, 2.32] | -4.81 | .130 |
| Funnel | | | | | | | | |
| Mathematics |  |  |  |  |  |  |  |  |
| AC-TC | 9 | 45 | 1.85 | 0.00 | 0.00 | [0.00, 0.01] | 2.58 | .133 |
| AC+TC | 16 | 72 | 1.62 | 0.00 | 0.00 | [-0.02, 0.01] | -1.46 | .308 |
| RT+TC | 13 | 56 | 3.84 | 0.00 | 0.01 | [-0.04, 0.04] | -0.28 | .797 |
| Computation |  |  |  |  |  |  |  |  |
| AC-TC | 5 | 13 | 2.04 | 0.00 | 0.00 | [0.00, 0.01] | 2.62 | .118 |
| AC+TC | 8 | 26 | 1.58 | -0.01 | 0.00 | [-0.03, 0.02] | -1.43 | .317 |
| RT+TC | 3 | 6 | 1.00 | 0.01 | 0.03 | [-0.43, 0.46] | 0.35 | .785 |
| Calculation |  |  |  |  |  |  |  |  |
| AC-TC | 5 | 13 | 2.04 | 0.00 | 0.00 | [0.00, 0.01] | 2.18 | .159 |
| Fact retrieval |  |  |  |  |  |  |  |  |
| AC+TC | 7 | 22 | 1.57 | -0.01 | 0.00 | [-0.03, 0.01] | -1.68 | .268 |
| Math reasoning |  |  |  |  |  |  |  |  |
| AC-TC | 3 | 3 | 1.00 | 0.00 | 0.00 | [-0.03, 0.03] | -1.05 | .486 |
| Number sense |  |  |  |  |  |  |  |  |
| AC-TC | 6 | 29 | 1.88 | -0.02 | 0.01 | [-0.09, 0.05] | -1.34 | .320 |
| AC+TC | 13 | 46 | 6.72 | -0.01 | 0.01 | [-0.04, 0.02] | -0.75 | .480 |
| RT+TC | 12 | 50 | 4.50 | -0.01 | 0.01 | [-0.04, 0.01] | -1.18 | .297 |
| Number word knowledge |  |  |  |  |  |  |  |  |
| AC-TC | 4 | 8 | 1.04 | -0.04 | 0.01 | [-0.20, 0.13] | -2.47 | .237 |
| Quantity processing |  |  |  |  |  |  |  |  |
| AC+TC | 6 | 25 | 1.80 | -0.02 | 0.00 | [-0.02, -0.01] | -8.51 | .018 |
| RT+TC | 4 | 22 | 1.58 | 0.01 | 0.00 | [0.00, 0.02] | 8.95 | .024 |
| Quantity-number linking |  |  |  |  |  |  |  |  |
| AC+TC | 7 | 9 | 2.01 | 0.00 | 0.02 | [-0.09, 0.09] | 0.00 | 1,000 |
| RT+TC | 7 | 12 | 2.46 | -0.01 | 0.00 | [-0.02, 0.00] | -2.52 | .105 |
| Numerical relations |  |  |  |  |  |  |  |  |
| AC-TC | 6 | 19 | 1.90 | -0.01 | 0.02 | [-0.09, 0.06] | -0.84 | .495 |
| AC+TC | 5 | 12 | 1.79 | -0.02 | 0.01 | [-0.08, 0.05] | -1.31 | .333 |
| RT+TC | 5 | 16 | 1.94 | -0.03 | 0.01 | [-0.08, 0.03] | -2.33 | .149 |
| Reading and writing |  |  |  |  |  |  |  |  |
| AC-TC | 3 | 3 | 1.00 | 0.00 | 0.00 | [-0.02, 0.02] | -0.11 | .933 |
| AC+TC | 6 | 20 | 1.22 | 0.00 | 0.00 | [-0.04, 0.03] | -1.02 | .469 |
| RT+TC | 3 | 8 | 1.00 | -0.01 | 0.01 | [-0.19, 0.17] | -0.56 | .677 |
| Phonetic coding |  |  |  |  |  |  |  |  |
| AC-TC | 3 | 3 | 1.00 | 0.00 | 0.00 | [-0.02, 0.02] | -0.09 | .940 |
| Retrieval fluency |  |  |  |  |  |  |  |  |
| AC+TC | 5 | 15 | 1.18 | 0.00 | 0.00 | [-0.01, 0.01] | -1.71 | .309 |
| RT+TC | 3 | 8 | 1.00 | -0.01 | 0.01 | [-0.19, 0.17] | -0.56 | .677 |
| Naming facility |  |  |  |  |  |  |  |  |
| AC+TC | 5 | 13 | 1.16 | 0.00 | 0.00 | [-0.01, 0.01] | -1.14 | .436 |
| RT+TC | 3 | 8 | 1.00 | -0.01 | 0.01 | [-0.20, 0.18] | -0.67 | .623 |
| Short-term working memory |  |  |  |  |  |  |  |  |
| AC-TC | 16 | 83 | 1.15 | 0.00 | 0.00 | [-0.01, 0.01] | 0.55 | .671 |
| AC+TC | 5 | 11 | 2.48 | 0.00 | 0.01 | [-0.03, 0.02] | -0.46 | .685 |
| RT+TC | 5 | 16 | 2.06 | 0.01 | 0.01 | [-0.02, 0.03] | 1.09 | .388 |
| Auditory short-term storage |  |  |  |  |  |  |  |  |
| AC-TC | 9 | 30 | 1.48 | 0.00 | 0.00 | [-0.01, 0.01] | -1.70 | .273 |
| Visual-spatial short-term storage |  |  |  |  |  |  |  |  |
| AC-TC | 9 | 24 | 3.41 | -0.02 | 0.02 | [-0.07, 0.03] | -0.91 | .425 |
| Working memory capacity |  |  |  |  |  |  |  |  |
| AC-TC | 13 | 27 | 1.72 | 0.00 | 0.00 | [0.00, 0.00] | 2.36 | .163 |
| Attentional control |  |  |  |  |  |  |  |  |
| AC+TC | 5 | 11 | 2.47 | 0.00 | 0.01 | [-0.03, 0.02] | -0.45 | .688 |
| RT+TC | 5 | 16 | 2.06 | 0.01 | 0.01 | [-0.02, 0.03] | 1.09 | .388 |
| Inhibition |  |  |  |  |  |  |  |  |
| AC+TC | 4 | 7 | 1.84 | 0.00 | 0.00 | [-0.02, 0.02] | -0.22 | .847 |
| RT+TC | 5 | 13 | 2.13 | 0.01 | 0.01 | [-0.01, 0.03] | 1.39 | .293 |
| Reaction and decision speed |  |  |  |  |  |  |  |  |
| RT+TC | 3 | 3 | 1.00 | -0.02 | 0.04 | [-0.47, 0.44] | -0.45 | .732 |
| Fluid reasoning |  |  |  |  |  |  |  |  |
| AC-TC | 3 | 5 | 1.00 | 0.00 | 0.00 | [0.00, 0.00] | 3.29 | .188 |
| IQ | | | | | | | | |
| Mathematics |  |  |  |  |  |  |  |  |
| AC-TC | 7 | 38 | 2.84 | 0.43 | 0.11 | [0.06, 0.80] | 3.81 | .035 |
| AC+TC | 14 | 66 | 3.58 | 0.01 | 0.18 | [-0.51, 0.53] | 0.04 | .969 |
| RT+TC | 12 | 53 | 2.05 | -0.13 | 0.23 | [-1.10, 0.84] | -0.56 | .631 |
| Computation |  |  |  |  |  |  |  |  |
| AC-TC | 3 | 10 | 1.00 | 0.66 | 0.03 | [0.23, 1.09] | 19.46 | .033 |
| AC+TC | 6 | 23 | 2.06 | 0.20 | 0.19 | [-0.61, 1.01] | 1.03 | .409 |
| Calculation |  |  |  |  |  |  |  |  |
| AC-TC | 3 | 10 | 1.00 | 0.66 | 0.04 | [0.14, 1.18] | 16.28 | .039 |
| Fact retrieval |  |  |  |  |  |  |  |  |
| AC+TC | 5 | 20 | 1.86 | 0.12 | 0.38 | [-1.63, 1.87] | 0.32 | .780 |
| Math reasoning |  |  |  |  |  |  |  |  |
| Number sense |  |  |  |  |  |  |  |  |
| AC-TC | 5 | 26 | 2.11 | 0.48 | 0.15 | [-0.14, 1.10] | 3.16 | .081 |
| AC+TC | 11 | 43 | 3.10 | -0.12 | 0.28 | [-1.00, 0.77] | -0.41 | .708 |
| RT+TC | 11 | 49 | 1.70 | -0.17 | 0.19 | [-1.15, 0.80] | -0.92 | .471 |
| Number word knowledge |  |  |  |  |  |  |  |  |
| AC-TC | 4 | 8 | 1.07 | 0.44 | 0.07 | [-0.30, 1.18] | 6.56 | .086 |
| Quantity processing |  |  |  |  |  |  |  |  |
| AC+TC | 5 | 24 | 1.18 | 0.15 | 0.15 | [-1.19, 1.49] | 0.99 | .483 |
| RT+TC | 3 | 21 | 1.00 | -1.47 | 1.04 | [-14.75, 11.80] | -1.41 | .393 |
| Quantity-number linking |  |  |  |  |  |  |  |  |
| AC+TC | 6 | 8 | 2.22 | -0.40 | 0.67 | [-3.04, 2.24] | -0.59 | .607 |
| RT+TC | 7 | 12 | 1.57 | -0.24 | 0.08 | [-0.69, 0.20] | -3.12 | .121 |
| Numerical relations |  |  |  |  |  |  |  |  |
| AC-TC | 5 | 16 | 2.34 | 0.53 | 0.22 | [-0.31, 1.38] | 2.37 | .123 |
| AC+TC | 4 | 11 | 1.82 | 1.77 | 0.41 | [-0.18, 3.71] | 4.32 | .059 |
| RT+TC | 5 | 16 | 1.61 | -0.27 | 0.43 | [-2.60, 2.07] | -0.62 | .609 |
| Reading and writing |  |  |  |  |  |  |  |  |
| AC-TC | 3 | 3 | 1.00 | 0.09 | 0.07 | [-0.75, 0.93] | 1.36 | .404 |
| AC+TC | 6 | 20 | 1.59 | -0.58 | 0.49 | [-3.31, 2.16] | -1.17 | .388 |
| RT+TC | 3 | 8 | 1.00 | -0.17 | 0.41 | [-5.38, 5.05] | -0.40 | .756 |
| Phonetic coding |  |  |  |  |  |  |  |  |
| AC-TC | 3 | 3 | 1.00 | 0.08 | 0.07 | [-0.79, 0.96] | 1.22 | .437 |
| Retrieval fluency |  |  |  |  |  |  |  |  |
| AC-TC | 5 | 15 | 1.32 | -0.08 | 0.08 | [-0.68, 0.52] | -1.00 | .465 |
| AC+TC | 3 | 8 | 1.00 | -0.17 | 0.41 | [-5.40, 5.06] | -0.40 | .756 |
| Naming facility |  |  |  |  |  |  |  |  |
| AC+TC | 5 | 13 | 1.32 | -0.09 | 0.07 | [-0.61, 0.44] | -1.21 | .401 |
| RT+TC | 3 | 8 | 1.00 | -0.22 | 0.44 | [-5.78, 5.34] | -0.50 | .705 |
| Short-term working memory |  |  |  |  |  |  |  |  |
| AC-TC | 16 | 83 | 5.40 | 0.17 | 0.14 | [-0.19, 0.53] | 1.21 | .277 |
| AC+TC | 5 | 11 | 1.59 | -0.14 | 0.14 | [-0.94, 0.67] | -0.95 | .465 |
| RT+TC | 4 | 14 | 1.24 | -0.18 | 0.61 | [-5.18, 4.83] | -0.29 | .812 |
| Auditory short-term storage |  |  |  |  |  |  |  |  |
| AC-TC | 9 | 30 | 4.10 | 0.05 | 0.22 | [-0.56, 0.67] | 0.24 | .824 |
| Visual-spatial short-term storage |  |  |  |  |  |  |  |  |
| AC-TC | 9 | 24 | 3.45 | 0.61 | 0.22 | [-0.05, 1.27] | 2.73 | .062 |
| Working memory capacity |  |  |  |  |  |  |  |  |
| AC-TC | 13 | 27 | 4.46 | 0.18 | 0.22 | [-0.40, 0.76] | 0.81 | .458 |
| Attentional control |  |  |  |  |  |  |  |  |
| AC+TC | 5 | 11 | 1.58 | -0.14 | 0.14 | [-0.94, 0.67] | -0.94 | .468 |
| RT+TC | 4 | 14 | 1.23 | -0.18 | 0.61 | [-5.21, 4.85] | -0.29 | .813 |
| Inhibition |  |  |  |  |  |  |  |  |
| AC+TC | 4 | 7 | 1.40 | -0.07 | 0.09 | [-0.66, 0.52] | -0.75 | .560 |
| RT+TC | 4 | 11 | 1.24 | -0.14 | 0.55 | [-4.65, 4.37] | -0.25 | .835 |
| Reaction and decision speed |  |  |  |  |  |  |  |  |
| RT+TC | 3 | 3 | 1.00 | -0.40 | 0.02 | [-0.59, -0.21] | -26.15 | .024 |
| Fluid reasoning |  |  |  |  |  |  |  |  |
| AC-TC | 3 | 5 | 1.00 | 0.41 | 0.18 | [-1.82, 2.63] | 2.33 | .258 |
| Reading | | | | | | | | |
| Mathematics |  |  |  |  |  |  |  |  |
| AC-TC | 7 | 39 | 2.61 | 1.01 | 0.39 | [-0.33, 2.35] | 2.62 | .091 |
| AC+TC | 11 | 56 | 3.24 | 0.10 | 0.19 | [-0.47, 0.67] | 0.54 | .623 |
| RT+TC | 10 | 44 | 1.83 | -0.54 | 0.22 | [-1.60, 0.51] | -2.42 | .148 |
| Computation |  |  |  |  |  |  |  |  |
| AC-TC | 3 | 11 | 1.00 | 0.69 | 0.12 | [-0.86, 2.23] | 5.64 | .112 |
| AC+TC | 5 | 22 | 2.26 | 0.43 | 0.17 | [-0.24, 1.11] | 2.49 | .116 |
| Calculation |  |  |  |  |  |  |  |  |
| AC-TC | 3 | 11 | 1.00 | 0.68 | 0.09 | [-0.52, 1.87] | 7.20 | .088 |
| Fact retrieval |  |  |  |  |  |  |  |  |
| AC+TC | 4 | 19 | 1.99 | 0.49 | 0.19 | [-0.34, 1.32] | 2.54 | .127 |
| Math reasoning |  |  |  |  |  |  |  |  |
| Number sense |  |  |  |  |  |  |  |  |
| AC-TC | 5 | 26 | 1.74 | 1.14 | 0.42 | [-0.94, 3.23] | 2.72 | .130 |
| AC+TC | 9 | 34 | 1.97 | 0.04 | 0.23 | [-0.98, 1.06] | 0.17 | .879 |
| RT+TC | 10 | 41 | 1.90 | -0.56 | 0.20 | [-1.46, 0.33] | -2.86 | .110 |
| Number word knowledge |  |  |  |  |  |  |  |  |
| AC-TC | 4 | 8 | 1.43 | 0.96 | 0.36 | [-1.34, 3.26] | 2.68 | .162 |
| Quantity processing |  |  |  |  |  |  |  |  |
| AC+TC | 4 | 16 | 1.38 | 0.47 | 0.28 | [-1.46, 2.39] | 1.66 | .292 |
| Quantity-number linking |  |  |  |  |  |  |  |  |
| AC+TC | 5 | 7 | 1.59 | -0.15 | 0.13 | [-0.86, 0.55] | -1.20 | .379 |
| RT+TC | 7 | 12 | 3.16 | 0.01 | 0.59 | [-1.82, 1.84] | 0.01 | .990 |
| Numerical relations |  |  |  |  |  |  |  |  |
| AC-TC | 5 | 16 | 2.05 | 1.53 | 0.49 | [-0.52, 3.58] | 3.13 | .086 |
| AC+TC | 4 | 11 | 1.17 | 0.32 | 1.02 | [-8.92, 9.55] | 0.31 | .802 |
| RT+TC | 5 | 16 | 1.46 | -0.59 | 0.19 | [-1.76, 0.58] | -3.17 | .128 |
| Reading and writing |  |  |  |  |  |  |  |  |
| AC-TC | 3 | 3 | 1.00 | 0.20 | 0.07 | [-0.76, 1.15] | 2.61 | .233 |
| AC+TC | 6 | 20 | 1.85 | -0.76 | 0.54 | [-3.29, 1.77] | -1.39 | .308 |
| RT+TC | 3 | 8 | 1.00 | -0.02 | 0.78 | [-9.88, 9.84] | -0.03 | .983 |
| Phonetic coding |  |  |  |  |  |  |  |  |
| AC-TC | 3 | 3 | 1.00 | 0.19 | 0.08 | [-0.85, 1.23] | 2.34 | .257 |
| Retrieval fluency |  |  |  |  |  |  |  |  |
| AC+TC | 5 | 15 | 1.44 | -0.43 | 0.32 | [-2.46, 1.61] | -1.34 | .353 |
| RT+TC | 3 | 8 | 1.00 | -0.02 | 0.77 | [-9.85, 9.81] | -0.03 | .982 |
| Naming facility |  |  |  |  |  |  |  |  |
| AC+TC | 5 | 13 | 1.44 | -0.47 | 0.38 | [-2.90, 1.97] | -1.23 | .382 |
| RT+TC | 3 | 8 | 1.00 | -0.06 | 0.89 | [-11.32, 11.20] | -0.07 | .957 |
| Short-term working memory |  |  |  |  |  |  |  |  |
| AC-TC | 15 | 82 | 5.30 | 0.60 | 0.25 | [-0.03, 1.24] | 2.41 | .058 |
| AC+TC | 5 | 11 | 1.82 | -0.11 | 0.24 | [-1.23, 1.00] | -0.49 | .679 |
| RT+TC | 4 | 14 | 1.83 | 0.75 | 0.49 | [-1.54, 3.04] | 1.53 | .276 |
| Auditory short-term storage |  |  |  |  |  |  |  |  |
| AC-TC | 9 | 30 | 4.06 | 0.53 | 0.22 | [-0.08, 1.14] | 2.38 | .075 |
| Visual-spatial short-term storage |  |  |  |  |  |  |  |  |
| AC-TC | 8 | 23 | 3.16 | 1.13 | 0.42 | [-0.17, 2.44] | 2.69 | .070 |
| Working memory capacity |  |  |  |  |  |  |  |  |
| AC-TC | 13 | 27 | 2.24 | 0.44 | 0.22 | [-0.40, 1.28] | 2.03 | .166 |
| Attentional control |  |  |  |  |  |  |  |  |
| AC+TC | 5 | 11 | 1.83 | -0.11 | 0.24 | [-1.23, 1.00] | -0.47 | .686 |
| RT+TC | 4 | 14 | 1.83 | 0.75 | 0.49 | [-1.55, 3.04] | 1.53 | .277 |
| Inhibition |  |  |  |  |  |  |  |  |
| AC+TC | 4 | 7 | 1.86 | -0.22 | 0.15 | [-0.92, 0.48] | -1.45 | .292 |
| RT+TC | 4 | 11 | 1.80 | 0.83 | 0.35 | [-0.86, 2.52] | 2.36 | .156 |
| Reaction and decision speed |  |  |  |  |  |  |  |  |
| RT+TC | 3 | 3 | 1.00 | -1.56 | 0.74 | [-10.94, 7.81] | -2.12 | .281 |
| Fluid reasoning |  |  |  |  |  |  |  |  |
| AC-TC | 3 | 5 | 1.00 | 0.27 | 0.15 | [-1.69, 2.23] | 1.75 | .331 |
| Math | | | | | | | | |
| Mathematics |  |  |  |  |  |  |  |  |
| AC-TC | 7 | 25 | 1.56 | -0.17 | 0.07 | [-0.55, 0.21] | -2.55 | .159 |
| AC+TC | 13 | 65 | 3.09 | 0.01 | 0.20 | [-0.61, 0.63] | 0.05 | .961 |
| RT+TC | 12 | 53 | 4.66 | -0.18 | 0.13 | [-0.52, 0.16] | -1.40 | .223 |
| Computation |  |  |  |  |  |  |  |  |
| AC-TC | 3 | 7 | 1.00 | -0.22 | 0.18 | [-2.50, 2.05] | -1.24 | .432 |
| AC+TC | 6 | 23 | 2.72 | -0.31 | 0.17 | [-0.88, 0.25] | -1.89 | .165 |
| Calculation |  |  |  |  |  |  |  |  |
| AC-TC | 3 | 7 | 1.00 | -0.17 | 0.19 | [-2.60, 2.25] | -0.91 | .529 |
| Fact retrieval |  |  |  |  |  |  |  |  |
| AC+TC | 5 | 20 | 1.92 | -0.32 | 0.17 | [-1.09, 0.45] | -1.87 | .207 |
| Math reasoning |  |  |  |  |  |  |  |  |
| Number sense |  |  |  |  |  |  |  |  |
| AC-TC | 4 | 17 | 1.92 | -0.38 | 0.26 | [-1.56, 0.80] | -1.44 | .292 |
| AC+TC | 10 | 42 | 1.85 | 0.15 | 0.16 | [-0.61, 0.90] | 0.90 | .469 |
| RT+TC | 11 | 49 | 4.28 | -0.20 | 0.17 | [-0.66, 0.26] | -1.16 | .308 |
| Number word knowledge |  |  |  |  |  |  |  |  |
| AC-TC | 3 | 6 | 1.00 | -0.28 | 0.35 | [-4.78, 4.23] | -0.78 | .577 |
| Quantity processing |  |  |  |  |  |  |  |  |
| AC+TC | 5 | 24 | 2.06 | -0.36 | 0.09 | [-0.73, 0.02] | -4.01 | .054 |
| RT+TC | 3 | 21 | 1.00 | 0.33 | 0.13 | [-1.32, 1.98] | 2.52 | .241 |
| Quantity-number linking |  |  |  |  |  |  |  |  |
| AC+TC | 5 | 7 | 1.43 | 0.26 | 0.07 | [-0.17, 0.68] | 3.91 | .100 |
| RT+TC | 7 | 12 | 2.55 | 0.06 | 0.14 | [-0.43, 0.54] | 0.40 | .719 |
| Numerical relations |  |  |  |  |  |  |  |  |
| AC-TC | 4 | 10 | 1.93 | -0.29 | 0.43 | [-2.20, 1.63] | -0.66 | .578 |
| AC+TC | 4 | 11 | 1.12 | -0.50 | 0.30 | [-3.46, 2.47] | -1.64 | .327 |
| RT+TC | 5 | 16 | 1.65 | -0.52 | 0.40 | [-2.67, 1.63] | -1.29 | .349 |
| Reading and writing |  |  |  |  |  |  |  |  |
| AC-TC | 3 | 3 | 1.00 | -0.10 | 0.03 | [-0.53, 0.33] | -2.93 | .210 |
| AC+TC | 6 | 20 | 1.87 | -0.13 | 0.17 | [-0.89, 0.63] | -0.81 | .509 |
| RT+TC | 3 | 8 | 1.00 | -0.11 | 0.19 | [-2.50, 2.27] | -0.61 | .653 |
| Phonetic coding |  |  |  |  |  |  |  |  |
| AC-TC | 3 | 3 | 1.00 | -0.10 | 0.04 | [-0.56, 0.36] | -2.71 | .225 |
| Retrieval fluency |  |  |  |  |  |  |  |  |
| AC+TC | 5 | 15 | 1.48 | 0.06 | 0.01 | [-0.02, 0.14] | 4.73 | .072 |
| RT+TC | 3 | 8 | 1.00 | -0.11 | 0.19 | [-2.50, 2.27] | -0.61 | .651 |
| Naming facility |  |  |  |  |  |  |  |  |
| AC+TC | 5 | 13 | 1.47 | 0.06 | 0.01 | [0.02, 0.10] | 9.60 | .027 |
| RT+TC | 3 | 8 | 1.00 | -0.13 | 0.21 | [-2.75, 2.48] | -0.66 | .630 |
| Short-term working memory |  |  |  |  |  |  |  |  |
| AC-TC | 15 | 82 | 2.72 | 0.00 | 0.06 | [-0.20, 0.19] | -0.04 | .967 |
| AC+TC | 5 | 11 | 1.24 | 0.00 | 0.15 | [-1.20, 1.19] | -0.03 | .982 |
| RT+TC | 4 | 14 | 1.24 | -0.27 | 0.15 | [-1.47, 0.93] | -1.84 | .279 |
| Auditory short-term storage |  |  |  |  |  |  |  |  |
| AC-TC | 9 | 30 | 3.21 | 0.04 | 0.14 | [-0.39, 0.46] | 0.26 | .813 |
| Visual-spatial short-term storage |  |  |  |  |  |  |  |  |
| AC-TC | 9 | 24 | 1.37 | -0.09 | 0.10 | [-0.76, 0.58] | -0.91 | .495 |
| Working memory capacity |  |  |  |  |  |  |  |  |
| AC-TC | 12 | 26 | 2.61 | 0.14 | 0.12 | [-0.26, 0.55] | 1.20 | .327 |
| Attentional control |  |  |  |  |  |  |  |  |
| AC+TC | 5 | 11 | 1.23 | 0.00 | 0.15 | [-1.21, 1.20] | -0.02 | .987 |
| RT+TC | 4 | 14 | 1.24 | -0.27 | 0.15 | [-1.46, 0.92] | -1.85 | .277 |
| Inhibition |  |  |  |  |  |  |  |  |
| AC+TC | 4 | 7 | 1.18 | 0.02 | 0.10 | [-0.91, 0.95] | 0.21 | .867 |
| RT+TC | 4 | 11 | 1.21 | -0.25 | 0.19 | [-1.84, 1.34] | -1.36 | .375 |
| Reaction and decision speed |  |  |  |  |  |  |  |  |
| RT+TC | 3 | 3 | 1.00 | 0.56 | 0.35 | [-3.91, 5.03] | 1.59 | .357 |
| Fluid reasoning |  |  |  |  |  |  |  |  |
| AC-TC | 3 | 5 | 1.00 | 0.16 | 0.06 | [-0.62, 0.94] | 2.55 | .238 |

*Note.* HA = high-level ability; MA = medium-level ability; LA = low-level ability; ; *CI95* = 95% confidence interval; Egger = Egger’s tests (moderator: *SE*); Funnel = funnel plot test (moderator: *N*); IQ = difference in IQ performance between MD and TD group (moderator: effect size); Reading = difference in reading performance between MD and TD group (moderator: effect size), Math = severity of MD (moderator: effect size); ST = number of studies; *b* = change in effect size; *SE* = standard error; AC = accuracy; RT = response time; TC = time constraints.

^a^Subordination of outcome reflects level, i.e. high-level ability (no indent), medium-level ability (medium indent), and low-level ability (large indent). Only those outcomes are shown for which the analysis could be performed.
